# New Insights into Interactions between Mushroom Aegerolysins and Membrane Lipids

**DOI:** 10.3390/toxins16030143

**Published:** 2024-03-09

**Authors:** Larisa Lara Popošek, Nada Kraševec, Gregor Bajc, Urška Glavač, Matija Hrovatin, Žan Perko, Ana Slavič, Miha Pavšič, Kristina Sepčić, Matej Skočaj

**Affiliations:** 1 Department of Biology, Biotechnical Faculty, University of Ljubljana, Jamnikarjeva 101, 1000 Ljubljana, Slovenia; larisalara.poposek@bf.uni-lj.si (L.L.P.); gregor.bajc@bf.uni-lj.si (G.B.); urska.krzic@gmail.com (U.G.); mtj.hrvtn@gmail.com (M.H.); zan.perko7@gmail.com (Ž.P.); anaaaslavic@gmail.com (A.S.); kristina.sepcic@bf.uni-lj.si (K.S.); 2 Department of Molecular Biology and Nanobiotechnology, National Institute of Chemistry, Hajdrihova 19, 1000 Ljubljana, Slovenia; nada.krasevec@ki.si; 3 Faculty of Chemistry and Chemical Technology, University of Ljubljana, Večna pot 113, 1000 Ljubljana, Slovenia; miha.pavsic@fkkt.uni-lj.si

**Keywords:** aegerolysins, MACPF, membranes, lipids, fungi, pore, phosphatidic acid, cardiolipin

## Abstract

Aegerolysins are a family of proteins that recognize and bind to specific membrane lipids or lipid domains; hence they can be used as membrane lipid sensors. Although aegerolysins are distributed throughout the tree of life, the most studied are those produced by the fungal genus *Pleurotus*. Most of the aegerolysin-producing mushrooms code also for proteins containing the membrane attack complex/perforin (MACPF)-domain. The combinations of lipid-sensing aegerolysins and MACPF protein partners are lytic for cells harboring the aegerolysin membrane lipid receptor and can be used as ecologically friendly bioinsecticides. In this work, we have recombinantly expressed four novel aegerolysin/MACPF protein pairs from the mushrooms *Heterobasidion irregulare*, *Trametes versicolor*, *Mucidula mucida*, and *Lepista nuda*, and compared these proteins with the already studied aegerolysin/MACPF protein pair ostreolysin A6–pleurotolysin B from *P. ostreatus*. We show here that most of these new mushroom proteins can form active aegerolysin/MACPF cytolytic complexes upon aegerolysin binding to membrane sphingolipids. We further disclose that these mushroom aegerolysins bind also to selected glycerophospholipids, in particular to phosphatidic acid and cardiolipin; however, these interactions with glycerophospholipids do not lead to pore formation. Our results indicate that selected mushroom aegerolysins show potential as new molecular biosensors for labelling phosphatidic acid.

## 1. Introduction

The aegerolysin protein family (Pfam 06355) consists of proteins that share some common features: similar low molecular weights (15–20 kDa), low isoelectric points, and β-sandwich structures [1]. Although they can be found in all domains of life, they are especially widespread in fungi [1]. The majority of the so far sequenced fungi do not code for aegerolysins, indicating they are not essential proteins [1]. Most of the knowledge about aegerolysins derives from their representatives from the fungal genus *Pleurotus*. Pleurotolysin A (PlyA), ostreolysin A (OlyA), and ostreolysin A6 (OlyA6) from *P. ostreatus* and pleurotolysin A2 (PlyA2) and erylysin A (EryA) from *P. eryngii* are the most studied aegerolysins which share another common feature, a lipid-binding ability [1]. Early experimental data showed that *Pleurotus* aegerolysins bind to membrane lipid domains composed of sphingomyelin (SM) and cholesterol (Chol) that are characteristic for vertebrates [2,3]. However, in 2015 another high-affinity lipid receptor, the insect-specific ceramide phosphoethanolamine (CPE), was identified [4]. All of the so-far characterized aegerolysins from the fungal genus *Pleurotus* recognize and bind to membranes enriched in CPE, and EryA from *P. eryngii* is the only of these aegerolysins that does not bind to SM/Chol, but recognizes only CPE [4]. Recently, it was identified that EryA binds also to membranes containing cardiolipin (CL) [5], while OlyA6 recognizes a CPE analogue ceramide aminoethylphosphonate (CAEP) which is widely encountered in mollusks [6]. Since these fungal aegerolysins are non-toxic and non-lytic, they have been developed as probes for labelling these specific membrane lipids and/or lipid domains [7,8,9,10,11,12]. Besides coding for aegerolysins, *Pleurotus* genomes also code for membrane attack complex/perforin (MACPF) domain-containing partner proteins. Pleurotolysin B (PlyB) is a MACPF protein from *P. ostreatus*, and the 96% identical erylysin B (EryB) is a MACPF protein from *P. eryngii* [2,13]. It was shown that the combination of lipid-sensing aegerolysins and MACPF-containing protein partners leads to the formation of transmembrane pores and lysis of the cells containing aegerolysin lipid receptors [2,3,14,15]. Specifically, it was shown that SM/Chol sensing OlyA6, in combination with PlyB, can be used for in vitro selective elimination of cancer urothelial cells with increased amount of lipid rafts (SM/Chol membrane complexes) in their membranes [16]. Furthermore, as OlyA6/PlyB or other *Pleurotus* aegerolysin/PlyB protein mixtures are toxic to the larvae of Colorado potato beetle (*Leptinotarsa decemlineata)* and western corn rootworm (*Diabrotica virgifera virgifera)*, they have been proposed as new environmentally friendly bioinsecticides [14,15]. 

Since the majority of what we know so far about these mushroom aegerolysins derives from the representatives from the fungal genus *Pleurotus*, and since these proteins have great biotechnological potential, we have characterized four novel aegerolysins and their MACPF domain-containing protein partners produced by other mushroom genera. We have expressed and biochemically characterized these protein pairs produced by *Heterobasidion irregulare* (annosus root or butt rot), *Trametes versicolor* (turkey tail), *Mucidula mucida* (porcelain fungus), and *Lepista nuda* (wood blewit). *L. nuda* and *M. mucida* belong to the order Agaricales, as the *Pleurotus* mushrooms, and they are both saprotrophic. While *L. nuda* can be found on decaying leaf litter, *M. mucida* typically grows on dead beech wood and it is also weakly parasitic to these trees [17,18]. *M. mucida* usually outcompetes other fungi locally by producing anti-fungal metabolites called strobilurins [18]. *H. irregulare* belongs to the order Russulales [19]. It is an infectious and invasive species and important primary pathogen of conifer forests across the southeastern US [20]. *T. versicolor* belongs to the order Polyporales. It is a saprotrophic, white-rot fungus which degrades lignin in wood [21]. It is known for its anti-cancer metabolites and it is one of the best investigated medicinal mushrooms [22]. In this study, the lipid-binding specificities of aegerolysins from *L. nuda*, *M. mucida*, *H. irregulare*, and *T. versicolor*, and their ability to form transmembrane pores in concert with their MACPF-protein partners, were characterized and compared to the most studied mushroom aegerolysin OlyA6 and its cytolytic complex OlyA6/PlyB from *P. ostreatus*. The obtained data provided surprising new insights about the mechanisms of function of mushroom aegerolysins.

## 2. Results

### 2.1. Bioinformatic Analysis

**Figure 1:**
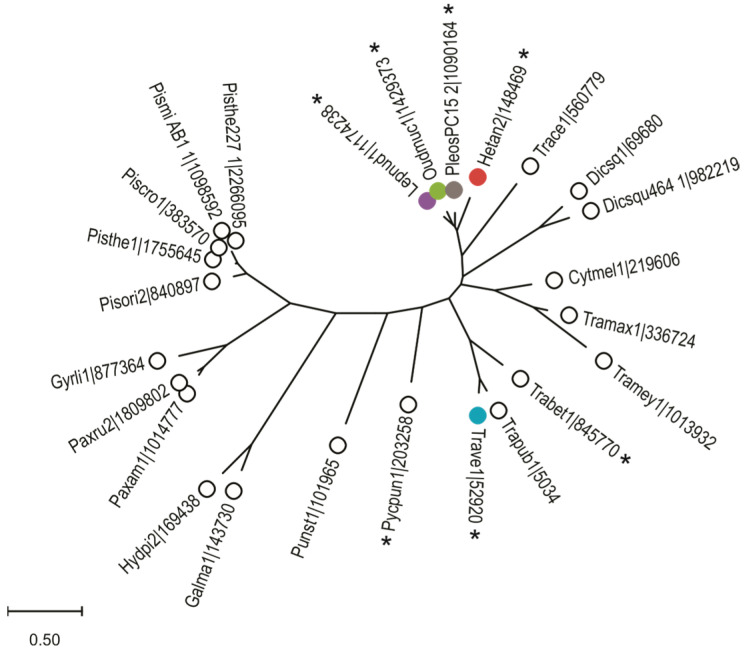
Phylogenetic analysis of aegerolysin singlets from Agaricomycetes. Aegerolysin singlets (32) from published fungal genomes identified in the JGI MycoCosm database [24] by Pfam protein domain PF06355 (Table S1). After Muscle alignment of the sequences (25), a maximum likelihood phylogenetic tree was inferred using the MEGA tools [26,27]. Aegerolysins from the selected fungal species are marked with closed circles: pleurotolysin A from *P. ostreatus* PC15 (PleosPC15_2|1090164, grey), heterolysin A from *H. irregulare* (Hetan2|148469, ruby), nudolysin A from *L. nuda* CBS 247.69 (Lepnud1|1174238, violet purple), versicolysin A from *T. versicolor* (Trave1|52920, deep teal), and mucolysin A from *M. mucida* (CBS 558.79, Oudmuc1|1429373, green). *, aegerolysin and MACPF gene pairs; MACPF, membrane attack complex/perforin.

Since *P. ostreatus* belongs to the class of Agaricomycetes, we searched this taxonomic group in the JGI MycoCosm fungal genome database [23,24] and found 491 of these genomes. The search for aegerolysins was performed using the Pfam protein domain PF06355. We identified 213 proteins encoded by 17% (85) of these 491 genomes. In the higher taxonomic group of Basidiomycota, which was covered by 639 genomes, only five aegerolysins were additionally found. Of these 85 genomes, 60 genomes are published with references and encoded for 160 proteins, which we further investigated (Table S1). They belong to species from different orders: Polyporales (22), Boletales (17), Agaricales (16), Russulales (3), and one from Corticiales and Geastrales [25]. These genomes contain between 1 and 24 aegerolysins and most of them (32) have only one aegerolysin. Additionally, nine genomes encoded for two aegerolysins, seven genomes for three, six genomes for four, two genomes for six or seven aegerolysins, one genome 15 and one genome even 24 aegerolysins. Because of the small or too large size of these aegerolysin sequences, we omitted 21 sequences from alignment to infer a phylogenetic tree (Figure S1). An additional phylogenetic tree of aegerolysin singlets was generated derived from 25 species, including only one strain per species (Figure 1).

The number of MACPF-domain hits is likely only an estimate, as they are often incorrectly annotated due to the higher number of possible introns than average fungal genes, and are therefore more likely to be missed by the Pfam search. Three such cases were identified among MACPFs from this study, and these were manually annotated in the MycoCosm database (#, Table S1) [24]. Since some aegerolysin and MACPF-domain protein partners have been shown to form pairs already at DNA level, such as pleurotolysin A and B [28], we searched for such pairs and indeed identified seven of them (*, Table S1). However, the aegerolysin proteins of these pairs did not cluster all together in the inferred phylogenetic tree (Figure 1). Four such pairs, nudolysin A and B (NudA and B) from *Lepista nuda*, mucolysin A and B (MucA and B) from *Mucidula mucida*, heterolysin A and B (HetA and B) from *Heterobasidion irregulare*, and versicolysin A and B (VerA and B) from *Trametes versicolor* were selected in this study for comparison with the ostreolysin A6/pleurotolysin B pair (OlyA6/PlyB) from *P. ostreatus*. 

As can be seen from the amino acid residues colored in blue, these selected protein sequences of non-uniform taxonomic origin are quite diverse (Figure S3). However, their gene structures are rather conserved: *olyA6*, *plyA*, *hetA*, *nudA*, *verA*, and *mucA* contain an intron at a conserved position when looking at the protein level and *mucA* contains an additional intron (Figures S2 and S3A). The *plyB*, *hetB*, and *nudB* genes contain eight introns, *verB* nine, and *mucB* seven. Six of these introns are at the conserved position as viewed at the protein level (Figure S3B); the intron at the seventh conserved position is absent in *mucB*. *plyB*, *hetB*, *verB*, and *mucB* have additional introns in the N-terminal region, which is barely homologous, and *verB* has another one elsewhere (Figure S3B). All of the selected MACPF proteins contain a well-conserved 13 amino acid signature in the form: Y-G-X-V-F-R-X_5_-G-G (Figure S3B) [29]. 

**Figure 2:**
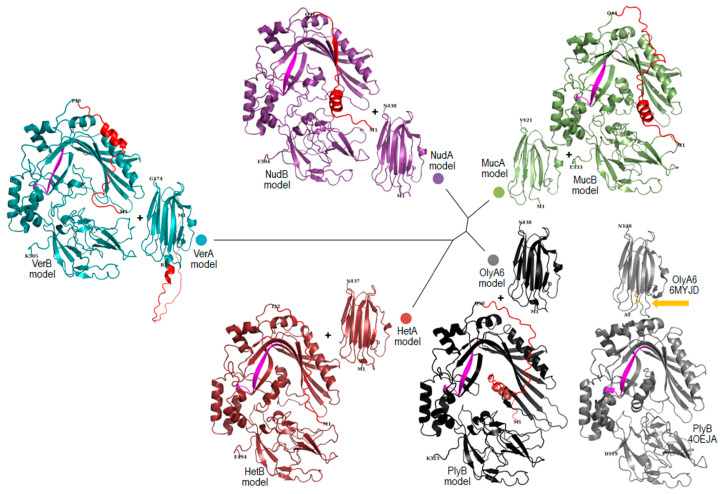
Five selected pairs of aegerolysins and MACPF proteins, their models, and structures. Phylogenetic tree of aegerolysins calculated by Mega [26,27]. Models of aegerolysins and protein partners calculated by AlphaFold2 [30]. Cartoon presentation by PyMOL [31]. Aegerolysins: OlyA6, ostreolysin A6 from *P. ostreatus*, model and structure PDB ID: 6MYJD, black and grey; HetA, heterolysin A from *H. irregulare*, ruby; NudA, nudolysin A from *L. nuda*, violet purple; VerA, versicolysin A from *T. versicolor*, deep teal; and MucA, mucolysin A from *M. mucida*, green. MACPF partner proteins: PlyB, pleurotolysin B, model, and structure PDB ID: 4OEJA, black and grey; HetB, heterolysin B, ruby; NudB, nudolysin B, violet purple; VerB, versicolysin B, deep teal; and MucB, mucolysin B, green. The position of the fragment of the chemical structure of SM (18:1) (in yellow–orange and marked with arrow). SM, sphingomyelin; typical signature Y/F-G-X_2_-F/Y-X_6_-G-G for the MACPF domain in fungi (magenta) [29]; N termini missing from the expression constructs are marked in red. Amino acids at N- and C-termini of the models and structures are numbered, as are the first amino acids after removal of the N-terminal. Structures and models of aegerolysins and MACPF proteins are not to scale; MACPF, membrane attack complex/perforin. For a detailed view of the SM fragment see OlyA6 = 6MYJD close-up in Figure 3A–C.

The models of the selected aegerolysins and MACPF proteins were generated using AlphaFold2 tool and compared with known structures PDB ID: 6MYJ chain D and 4OEJ chain A, respectively (Figure 2). Superposition of the generated models with the solved crystal structures shows the expected general overlap of β-sandwich or three-domain MACPF proteins, respectively. The MACPF signatures of PlyB, HetB, NudB, VerB, and MucB superimpose well and are located on a β-sheet in the center of molecule (Figure 2). The non-uniform N-terminal, which is excluded from the determinated PlyB structure PDB ID: 4OEJ, is modelled as a combination of a low-confidence α-helix and an unstructured chain. Similar observations can be made for other N-terminal chains of different lengths from selected MACPF protein models and for the longer N-terminal of VerA.

**Figure 3:**
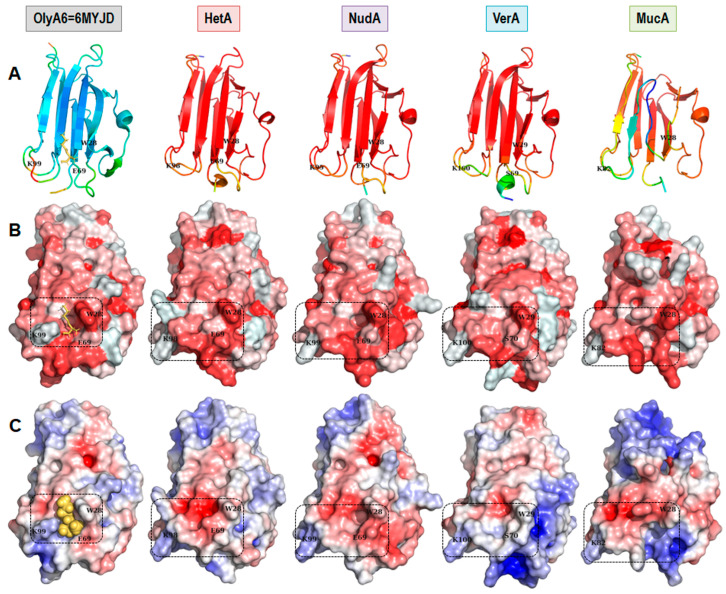
Comparison of the hydrophobicity and electrostatics of five selected aegerolysin. Models of aegerolysins calculated with AlphaFold2 [30,34]; visualization with PyMOL [31]. (**A**) The protein structure is validated by the B-factor; from red, over yellow and green to blue, from most to least flexible. The models by the predicted lDDT; red, most confidence. Cartoon representation. (**B**) Hydrophobicity according to the Eisenberg scale for hydrophobicity [35]; red, hydrophobic surface. (**C**) APBS electrostatics at pH 7 [36]; red, negative electrostatic potential; blue, positive surface (scale ranges from +/−5). The putative SM binding site, black dashed line; The position of the amino acid residues corresponding to W28, E69 (or equivalent), and K99 are labeled [32]. SM, sphingomyelin, yellow–orange sticks or spheres. Aegerolysins: OlyA6, ostreolysin A6 from *P. ostreatus* structure PDB ID: 6MYJD; HetA, heterolysin A from *H. irregulare*; NudA, nudolysin A from *L. nuda*; VerA, versicolysin A from *T. versicolor*; and MucA, mucolysin A from *M. mucida*; lDDT, local distance difference test; APBS, adaptive Poisson–Boltzmann solver.

The β-sandwich of the OlyA6 structure consists of eight β-strands and a one α-helix connected by four flexible loops at the C-terminal side and three flexible loops at the N-terminal side (Figure 3A). The crystal structure PDB ID: 6MYJ chain D (6MYJD) shows the lipid-binding site in OlyA6, W28, and K99, residues located at two separate loops at the N-terminus, form the boundaries of the shallow lipid-binding channel (Figure 3B,C) [32]. OlyA6 recognizes SM/Chol complexes in membranes and cholesterol specificity is determined by a single glutamic acid residue E69 [32]. Considering the high B factor/low predicted local distance difference test (lDDT), it appears that the loops at the C-terminus end are the most flexible part of the structure or the least reliable in the generated aegerolysin models (Figure 3A). K99 is one of the most flexible residues in the crystal structure PDB ID: 6MYJD. Due to the gap in the sequence of MucA, as shown in Figure S3 (as a consequence of an additional intron), the protein chain is shorter, which has a detrimental effect on the recognizable aegerolysin β-sandwich fold (Figure 3A); the absence of E69 appears to impair the lipid-binding channel (Figure 3B,C). Compared to other parts of the aegerolysin structures or structural models, the part expected to be involved in lipid binding appeared to be more hydrophobic (more red, Figure 3B). At the same time, the shallow channel of the putative SM binding sites appeared to have a more negative electrostatic potential, at least for HetA and NudA (more red, Figure 3C). These seemed to be in accordance with the situation in the structure of pleurotolysin A (PDB ID: 4OEB) from *P. ostreatus* which was crystallized without a lipid (not shown) [33]. VerA appeared to be more neutral (white) (Figure 3C). The adaptive Poisson–Boltzmann solver (APBS) electrostatics of the structural models could only be carried out at a default pH value of 7.0 and not at pH 6.0 or pH 8.0.

The sequences of expression of these proteins were taken from the database and were not cloned from mRNA. The N-terminal region of VerA is longer as compared to other aegerolysins and was later omitted during the cloning to gain its truncated version Δ37VerA; the variable N-terminal was also omitted from all selected MACPF sequences (Figure S3).

The native aegerolysins NudA and HetA have a similar calculated molecular mass to OlyA6 (≈15 kDa). MucA is shorter (≈13 kDa) and VerA is longer (≈19 kDa) due to the additional N-terminal peptide as compared to OlyA6. The truncated version of VerA, Δ37VerA, lacking N-terminal peptide has a calculated molecular similar as OlyA6 (Table S2). The calculated isoelectric points of OlyA6, NudA, and VerA are between 5.1 and 5.5, while those of MucA and HetA are just below 7.0. The deletion mutant Δ37VerA is the only aegerolysin in the study with a basic isoelectric point (7.7) (Table S2). NudA, MucA, and HetA are 75–80% identical to OlyA6 and among them NudA is the most identical to OlyA6 (≈80%). On the other hand, the amino acid identity between OlyA6 and/or VerA and Δ37VerA is only 55% (Table S2). The native truncated versions of their MACPF protein partners have similar calculated molecular weights (52.0–54.0 kDa) (Table S3). Their calculated isoelectric points are all acidic and below the calculated isoelectric point of PlyB (6.4), and among them the isoelectric point of VerB is the lowest (4.9) (Table S3). As in case of the studied aegerolysins and OlyA6, their MACPF protein partners also show similar degrees of amino acid identity with PlyB. NudB and MucB are ≈70% identical to PlyB, while HetB and VerB show ≈60% identity to PlyB (Table S3).

### 2.2. Production of Recombinant Proteins

**Figure 4:**
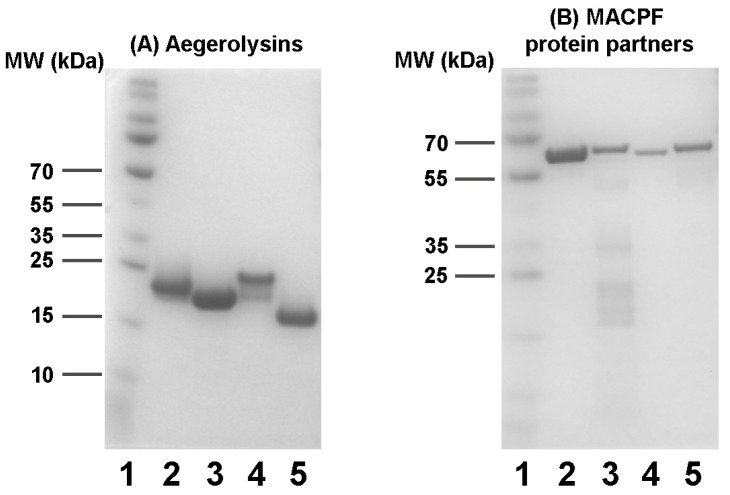
SDS-PAGE analysis of isolation of aegerolysins and their native MACPF proteins partners. (**A**) Lane 1: molecular weight marker; lane 2: ostreolysin A6 (OlyA6); lane 3: heterolysin A (HetA); lane 4: nudolysin A (NudA); lane 5: truncated version of versicolysin A (Δ37VerA). (**B**) Lane 1: molecular weight marker; lane 2: pleurotolysin B (PlyB); lane 3: nudolysin B (NudB); lane 4: versicolysin B (VerB); lane 5: mucolysin B (MucB). MW, molecular weight (kDa); MACPF, membrane attack complex/perforin.

While aegerolysins were isolated as soluble proteins, their MACPF protein partners were obtained from insoluble inclusion bodies. The expressed MACPF proteins PlyB (Δ48PlyB), NudB (Δ30NudB), HetB (Δ21HetB), VerB (Δ29VerB), and MucB (Δ43 MucB) were prepared in their truncated form, lacking N-terminal peptide, as already described for PlyB [3]. No signal peptides were predicted for these proteins, at least no standard secretion signal peptides that are transported by the Sec translocon and cleaved by signal peptidase I (Sec/SPI) [28,37] (Table S7). However, the presence or absence of a signal peptide does not say everything about the localisation of eukaryotic proteins. To find out more about the sorting of these proteins we made a prediction of subcellular localisation, which showed that these proteins would be located in the cytoplasm and in the extracellular space [38] (Table S8). The aegerolysins were typically isolated in 30–80 µM, and their MACPF protein partners in 1–15 µM concentrations. Although the DNA sequencing after cloning confirmed the sequences of HetB and MucA were correct, we have not been able to isolate these two proteins regardless of many attempts under a variety of growing conditions and using different recombinant strains. The aegerolysins NudA, HetA, VerA, and Δ37VerA, and their corresponding MACPF protein partners NudB, VerB, and MucB, were isolated using Ni^2+^-NTA affinity chromatography. As VerA with an atypical peptide at the N-terminal was unstable and prone to aggregation, we constructed and isolated its truncated version Δ37VerA. SDS-PAGE analysis of the isolated recombinant proteins is shown in Figure 4.

### 2.3. Membrane Binding Studies

#### 2.3.1. Sedimentation Assay

The interaction of purified recombinant aegerolysins with MLVs prepared from natural and commercial lipid mixtures was initially tested using a sedimentation assay at pH 8.0. We confirmed the previously reported data from a sedimentation assay by Panevska et al. [14], i.e., the binding of OlyA6 to equimolar MLVs composed of SM:Chol and CPE:POPC:Chol. As already reported [3,12,14], OlyA6 did also bind to vesicles made from lipids from RBC or Sf9 cells, but not to equimolar MLVs composed of POPC:Chol or to the other MLVs tested (Figure S4). While HetA showed similar behavior to OlyA6, NudA bound only to vesicles supplemented with CPE and to vesicles made of Sf9 cells, and had no affinity for the other vesicles tested (Figure S4). Δ37VerA did not bind to any of the vesicles tested (Figure S4). 

#### 2.3.2. Surface Plasmon Resonance

**Figure 5:**
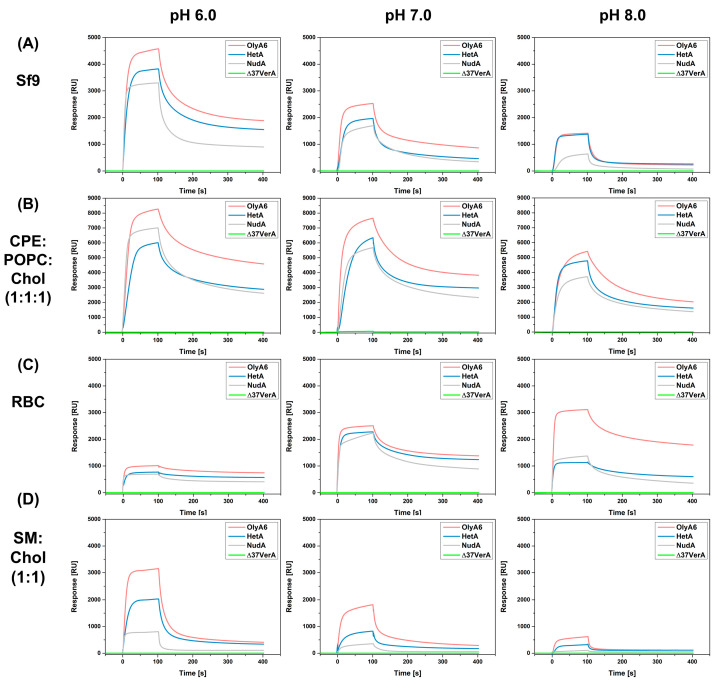
Surface plasmon resonance analysis of interaction of aegerolysins with LUVs containing aegerolysin sphingolipid receptors. Large unilamellar vesicles (LUVs) were composed of (**A**) total lipids extracted from Sf9 cells, (**B**) CPE:POPC:Chol (1:1:1, mol:mol:mol), (**C**) total lipid extracted from RBC, (**D**) SM:Chol (1:1, mol:mol) and immobilized on a Biacore L1 chip to 1.000–12.000 RU. The analytes (OlyA6, HetA, NudA, Δ37VerA) at a 5 µM concentration were injected at a flow rate of 10 μL/min with an association time of 1 min in the running buffer (pH 6.0, 7.0 or 8.0) at 25 °C. Representative sensorgrams of triplicate experiments, in which the standard error did not exceed 5%, are shown. OlyA6, ostreolysin A6; HetA, heterolysin A; NudA, nudolysin A; Δ37VerA, deletion mutant of versicolysin A; Sf9, lipid isolate of Spodoptera frugiperda Sf21 cells; RBC, lipid isolate from bovine erythrocytes; CPE, ceramide phosphoethanolamine; POPC, 1-palmitoyl-2-oleoyl-sn-glycero-3-phosphocholine; RBC, bovine red blood cells; SM, sphingomyelin; Chol, cholesterol; RU, response units.

**Figure 6:**
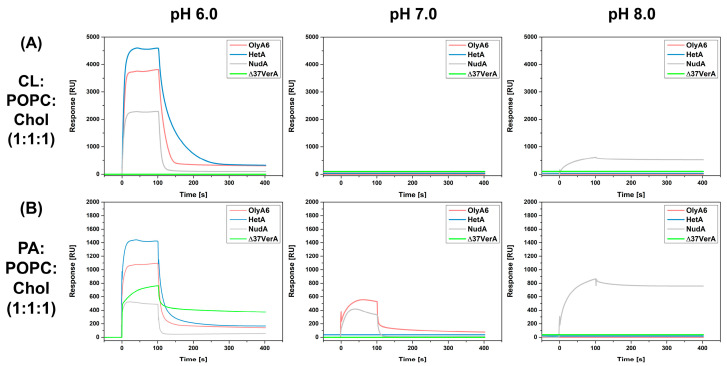
Surface plasmon surface analysis of interaction of aegerolysins with LUVs containing aegerolysin glicerophospholipid receptors. Large unilamellar vesicles (LUVs) containing CL (**A**) or (**B**) PA were immobilized on a Biacore L1 chip to 3.500–9.000 RU and analytes (OlyA6, NudA, HetA, and Δ37VerA in the 5 µM concentration) injected at a flow rate of 10 μL/min with an association time of 1 min in a running buffer (pH 6.0, 7.0, or 8.0) at 25 °C. Representative sensorgrams of triplicate experiments, in which the standard error did not exceed 5%, are shown. OlyA6, ostreolysin A6; HetA, heterolysin A; NudA, nudolysin A; Δ37VerA, deletion mutant of versicolysin A; CL, cardiolipin; Chol, cholesterol; PA, phosphatidic acid; POPC; 1-palmitoyl-2-oleoyl-*sn*-glycero-3-phosphocholine; RU, response unit.

**Figure 7:**
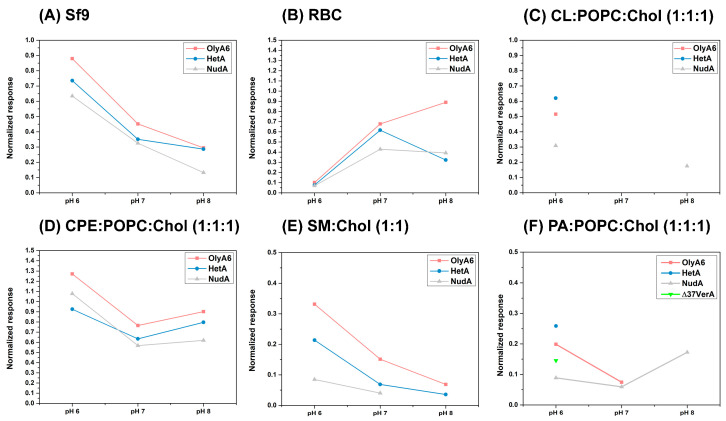
pH-dependence of aegerolysin affinities for LUVs of different compositions. The amounts of bound aegerolysins in RUs at 100 s were normalized with the amount of immobilized large unilamellar vesicles (LUVs) in RUs. OlyA6, ostreolysin A6; HetA, heterolysin A; NudA, nudolysin A; Sf9, lipid isolate of *Spodoptera frugiperda* Sf21 cells; RBC, lipid isolate from bovine red blood cells; CL, cardiolipin; CPE, ceramide phosphoethanolamine; SM, sphingomyelin; Chol, cholesterol; PA, phosphatidic acid; POPC; 1-palmitoyl-2-oleoyl-*sn*-glycero-3-phosphocholine; RU, response unit.

SPR studies were conducted to follow the interactions of investigated aegerolysins OlyA6, NudA, HetA, and Δ37VerA (5 µM) with on-chip immobilized LUVs (Figure 5 and Figure 6). The stability and the quantity of immobilized vesicles were strongly influenced by pH value. Since we aimed to investigate at which pH value the interaction between the aegerolysins and the vesicles is the strongest, we normalized the RU responses at 100 s for each aegerolysin with the quantity of immobilized vesicles (in RUs), and the obtained graphs are shown in Figure 7. The quantites of immobilized vesicles in RUs in each experiment are shown in Table S4. Firstly, we tested the binding of these aegerolysins to lipid membranes for which the interactions were already documented [3,12,14,39], like the membranes reconstituted from total lipids of Sf9 cell and RBC or membranes composed of SM:Chol (1:1, mol:mol) or CPE:POPC:Chol (1:1:1, mol:mol:mol).

The binding of OlyA6 to lipids extracted from Sf9 cell was the strongest, while the binding of NudA was the weakest at all three tested pH values (Figure 5A). HetA showed less affinity for Sf9 lipids compared to OlyA6 at pH 6.0 and 7.0; however, at pH 8.0 the binding affinity was almost identical. The binding of all three proteins (OlyA6, NudA, and HetA) to lipids extracted from Sf9 was the most efficient at pH 6.0 and the weakest at pH 8.0 (Figure 7A). Sfingolipid CPE is a constituent of Sf9 cell membranes. In a complex with Chol, it is a high-affinity receptor for the majority of the thus far characterized fungal aegerolysins [4,14,39]. As in the case of lipids from Sf9 cells, OlyA6, NudA, and HetA showed strong affinity for the CPE-containing lipid mixture and, among them, OlyA6 had the highest affinity at all three tested pH values (Figure 5B). OlyA6, NudA, and HetA showed the strongest binding to CPE-containing vesicles at pH 6.0 (Figure 7D). Δ37VerA did not bind to CPE/Chol lipid complexes or to lipids from Sf9 cells. 

OlyA6, NudA, and HetA bound to lipids extracted from RBC and their affinity was comparable at pH 6.0 and 7.0 (Figure 5C). However, at pH 8.0, OlyA6 shows substantially higher affinity for RBC lipids compared to NudA and HetA. The normalization of the RU response showed that the binding of OlyA6, NudA, and HetA is weakest at pH 6.0 (Figure 7B). The binding of OlyA6 to RBC lipids was strongest at pH 8.0, while HetA and NudA showed the strongest binding at pH 7.0. Δ37VerA did not bind to lipids extracted from RBC. OlyA6, NudA, and HetA showed a strong pH-dependent binding to SM/Chol lipid membranes and their binding was strongest at pH 6.0 (Figure 7E). Furthermore, at all three pH values tested, OlyA6 showed the strongest binding (Figure 5D). While NudA did not bind to SM/Chol lipid membranes at pH 8.0, Δ37VerA had no affinity for this lipid mixture at any pH value tested.

Next, we evaluated the binding of OlyA6, NudA, HetA, and Δ37VerA to LUVs containing equimolar ratios of POPC, cholesterol, and various glycerophospholipids, e.g., CL, PS, PE, or PA, as well as to POPC:Chol (1:1, mol:mol) which served as negative control. None of the four proteins bound to PE or PS-containing lipid mixtures nor to POPC:Chol (1:1, mol:mol)) at any pH value tested (Figure S5). HetA, OlyA6, and NudA showed very strong binding to CL-containing vesicles at pH 6.0 and, among them, HetA showed the strongest affinity (Figure 6A). While none of the tested aegerolysins had an affinity for CL-containing membranes at pH 7.0, NudA showed some binding to this lipid mixture at pH 8.0 (Figure 6A). HetA, OlyA6, Δ37VerA, and NudA also had a strong affinity for PA-containing vesicles at pH 6.0 and, among them, HetA again showed the strongest affinity (Figure 6B). Of all four aegerolysins, Δ37VerA showed the strongest, albeit reversible, association with PA-containing vesicles at pH 6.0. Furthermore, since Δ37VerA does not associate with other membrane lipids, one can conclude that Δ37VerA is a specific probe for labelling PA at pH 6.0. OlyA6 and NudA showed some affinity for PA-containing lipids at pH 7.0 and only NudA bound to PA-containing vesicles at pH 8.0. While the best binding of HetA and OlyA6 to PA-containing vesicles occurred at pH 6.0, this was not the case for NudA, which showed the highest affinity for this lipid mixture at pH 8.0 (Figure 7F).

### 2.4. Membrane Permeabilization Studies

#### 2.4.1. Calcein Release Assay

**Figure 8:**
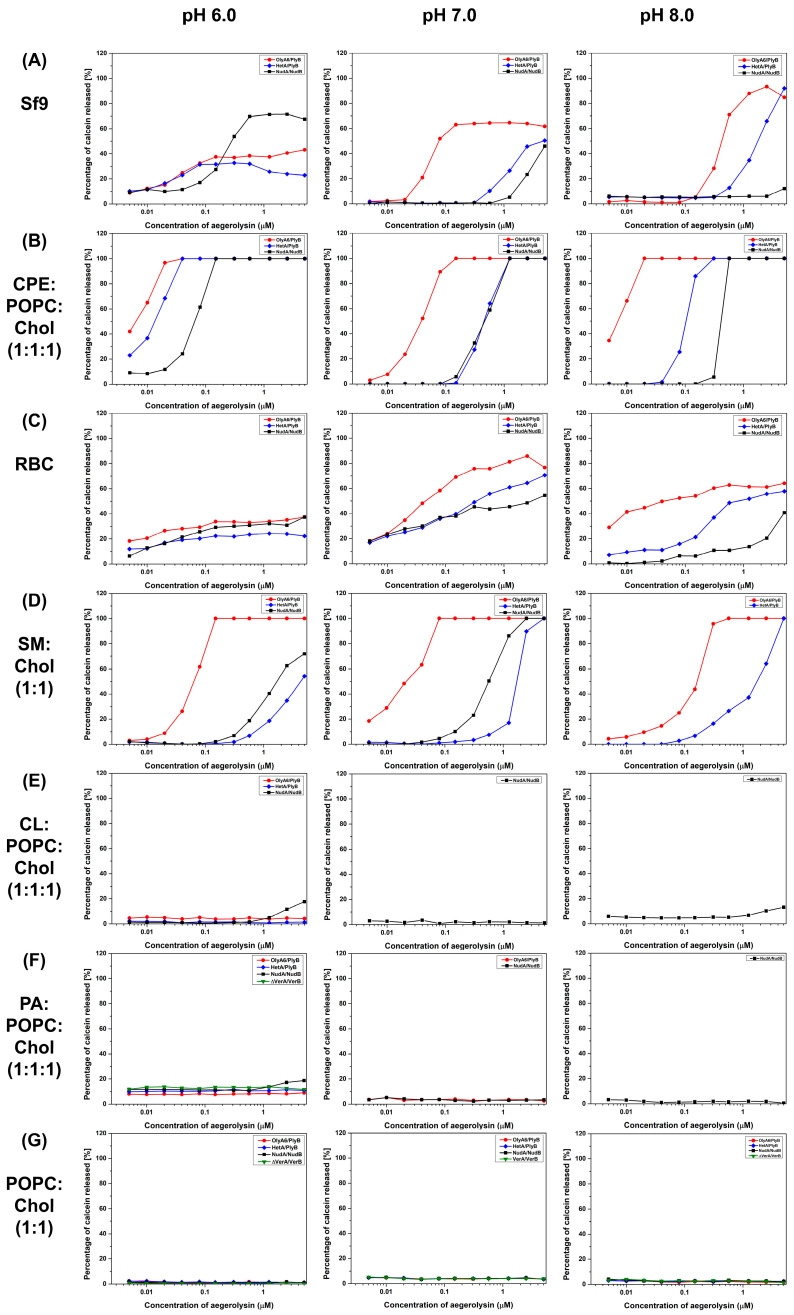
Permeabilization of SUVs of various lipid compositions by mushroom aegerolysin/MACPF protein complexes OlyA6/PlyB, NudA/NudB, Δ37VerA/VerB, and HetA/PlyB. Fluorescence intensity of calcein released from small unilamellar vesicles (SUVs) of various lipid compositions (as indicated), prepared in calcein buffer (pH 6.0, 7.0 or 8.0), was monitored as described in Materials and Methods. OlyA6, ostreolysin A6; PlyB, pleurotolysin B; HetA, heterolysin A; NudA, nudolysin A; NudB, nudolysin B; Δ37VerA, deletion mutant of versicolysin A; VerB, versicolysin B; Sf9, lipid isolate of *Spodoptera frugiperda* Sf21 cells; RBC, lipid isolate from bovine erythrocyte; POPC; 1-palmitoyl-2-oleoyl-*sn*-glycero-3-phosphocholine, CPE, ceramide phosphoethanolamine; SM, sphingomyelin; Chol, cholesterol; CL, cardiolipin; PA, phosphatidic acid; MACPF, membrane attack complex/perforin.

We evaluated the permeabilization of the protein mixtures of aegerolysins and MACPF protein partners only for those membranes for which the SPR experiments showed positive results. The permeabilization of SUVs containing SM or CPE, or SUVs reconstituted from total lipids extracted from RBC or Sf9 cells by OlyA6/PlyB, NudA/NudB, or HetA/PlyB complexes, was concentration-dependent (Figure 8). Since we were not able to obtain HetB, PlyB was used as a protein partner together with HetA. In most of these experiments, OlyA6/PlyB exhibited the highest membrane-disrupting potential. An exception are SUVs prepared from Sf9 cells at pH 6.0, which were most efficiently permeabilized by the NudA/NudB protein pair. Surprisingly, no lytic activity was observed when the tested aegerolysin/MACPF complexes (OlyA6/PlyB, NudA/NudB, HetA/PlyB, or Δ37VerA/VerB) were incubated with equimolar vesicles composed of CL:POPC:Chol and PA:POPC:Chol, despite the high affinity of the aegerolysin protein partners for these lipid mixtures (Figure 6A,B).

#### 2.4.2. Hemolytic Activity

The mixtures of each of the aegerolysins and their corresponding native MACPF protein partners (OlyA6/PlyB, NudA/NudB, and Δ37VerA/VerB) were assayed for hemolytic activity at different pH values (6.0, 7.0, and 8.0) (Table 1). Δ37VerA/VerB did not show any hemolytic activity at any pH tested, while OlyA6/PlyB showed the strongest hemolytic activity at all three pH values. The hemolytic activity of OlyA6/PlyB and NudA/NudB was the fastest at pH 6.0. We also tested the hemolytic activity of MucB in combination with OlyA6 and NudA, but no hemolysis occurred. Hemolytic activity was also detected with the HetA/PlyB protein mixture, which was also most active at pH 6.0. However, protein pair HetA/PlyB was less active compared to the OlyA6/PlyB protein mixture at all pH values tested (Table 1). Aegerolysins (1 µM) or MACPF protein partners (0.1 µM) alone were not hemolytic at pH values of 6.0, 7.0, or 8.0 (Figure S6).

#### 2.4.3. Sf9 Cytotoxicity Studies

**Figure 9:**
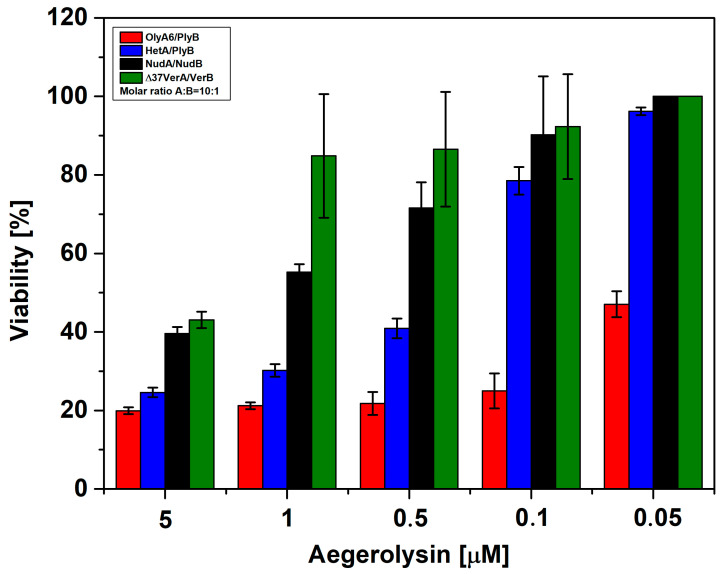
Effect of mushroom aegerolysin/MACPF protein mixtures on survival of Sf9 cells. Percentage of viability in % of Sf9 cells treated with OlyA6/PlyB, NudA/NudB, HetA/PlyB, and Δ37VerA/VerB is expressed as the ratio between the absorbance of treated cells and the absorbance of control cells × 100% after 1 h of exposure. Data are means ± SD from three independent experiments. OlyA6, ostreolysin A6; pleurotolysin B, PlyB; HetA, heterolysin A; NudA, nudolysin A; NudB, nudolysin B; Δ37VerA, deletion mutant of versicolysin A; VerB, versicolysin B; Sf9, lipid isolate of *Spodoptera frugiperda* Sf21 cells; MACPF, membrane attack complex/perforin.

According to the cell viability assay, all of the tested protein mixtures (OlyA6/PlyB, HetA/PlyB, NudA/NudB, and Δ37VerA/VerB) showed some extent of cytotoxicity towards the Sf9 cell line (Figure 9). However, there was a great variability in cytotoxicity among the tested protein pairs. Here again OlyA6/PlyB was the most toxic protein mixture at all of the tested concentrations. Δ37VerA/VerB severely reduced cell viability only at the highest concentration tested, while NudA/NudB was slightly more toxic than Δ37VerA/VerB, but much less toxic than the HetA/PlyB and OlyA6/PlyB protein pairs. When tested alone at a 5 µM concentration, OlyA6 decreased the viability of the cells by 60%, HetA by 40%, and NudA by 20% (Figure S7A). Δ37VerA and 0.5 µM of MACPF protein partners (PlyB, NudB, and VerB) did not reduce the viability of Sf9 cells (Figure S7A,B).

## 3. Discussion

Aegerolysin proteins were first discovered in the oyster mushrooms (*P. ostreatus*) in 1979 [40], and since then they have been the best studied representatives of this protein family. Recently, *Pleurotus* aegerolysins have gained considerable attention as tools for labeling specific lipids [11,12], or as environmentally friendly biopesticides [14,41]. Because of their promising biotechnological potential, it is important to further characterize them, and to study new aegerolysin-like proteins from other organisms. 

Our bioinformatic analyses demonstrated that approximately 20% of mushroom genomes belonging to the class Agaricomycetes encode for aegerolysins. We therefore aimed to recombinantly express and characterize four novel aegerolysins from Agaricomycetes, namely NudA from *L. nuda*, HetA from *H. irregulare,* MucA from *M. mucida*, and VerA from *T. versicolor*, as well as their native MACPF-partnering proteins NudB, HetB, MucB, and VerB. Since VerA aggregated after isolation and had an additional N-terminal sequence which is not characteristic for other aegerolysins, we constructed its truncated form, Δ37VerA, which was not prone to aggregation. Δ37VerA differs from all of the other aegerolysins studied so far [1] in having a basic rather than acidic isoelectric point. Further, the isolation of MucA was not successful, probably because of the shorter protein sequence resulting in impaired β-sheets. The MACPF protein partners of aegerolysins are expressed as pre-proteins and their N-terminals are later processed to gain functional proteins in producing mushrooms. For this reason, all MACPF proteins in this study were expressed in their truncated versions as was already demonstrated for PlyB [3]. In contrast to NudB, MucB, and VerB, the production of HetB was not successful despite several isolation protocols and different *E. coli* strains being assayed. 

Previously, it was demonstrated that the selected *Pleurotus* aegerolysins can be applied as probes for labeling cholesterol-sequestered membrane sphingolipids, namely SM in mammalian cells or CPE or CAEP in the cell membranes of insects and mollusks, respectively. We also showed that *Pleurotus* aegerolysins are lytic to sphingolipid-supplemented membranes when combined with the MACPF protein partner PlyB [3,6,12,14,15]. Glycerophospholipids were not regarded as aegerolysin lipid receptors until the recent study by Sakihara et al. [5], who demonstrated the binding of EryA to artificial lipid vesicles containing cardiolipin (CL) and its accumulation in CL-enriched membrane regions when expressed in *E. coli* in its fluorescent fusion form. We therefore performed a comprehensive analysis of the membrane-binding specificity of NudA, HetA, and Δ37VerA, and the membrane-permeabilizing activity of the NudA/NudB, HetA/PlyB, and Δ37VerA/VerB protein pairs, using lipid vesicles of various lipid compositions at three different pH values, and compared the results the to the activities of the well-characterized *Pleurotus* aegerolysin OlyA6 and its lytic complex OlyA6/PlyB. A sedimentation assay at pH 8.0 was initially used to confirm our previous results regarding the binding of OlyA6 to MLVs, which we used as a control aegerolysin throughout the study. None of the newly characterized mushroom aegerolysins reached the binding affinity of OlyA6 for sphingolipid (SM or CPE)-containing membranes. In the majority of cases, the strongest aegerolysin membrane interactions were achieved at pH 6.0, except with vesicles reconstituted from total RBC lipids; probably because of the instability of these vesicles at low pH values [42]. NudA and HetA, which share the highest (75–80%) degree of amino acid identity with OlyA6, also mirrored OlyA6 in their specificity for membrane lipid receptors and interacted with lipid vesicles supplemented with biological or commercial sphingolipids. However, this was not the case for Δ37VerA, which did not associate with membrane sphingolipids. Such behavior could derive from the fact that, in Δ37VerA, the negatively charged amino acid residue E69, which is involved in sphingolipid recognition [32], is replaced by a polar serine residue. Regarding the binding to glycerophospholipid-supplemented vesicles, our study confirmed the interaction of OlyA6, HetA, and NudA with membrane-associated CL. In contrast to EryA, which was able to bind CL-containing vesicles at pH 7.4 [5], the aegerolysins tested in this work were able to interact with these vesicles only at pH 6.0. A further step to gain an atomistic understanding of the different lipid binding could be to compare the affinities and side chains of the amino acids involved in the docking of CL or CPE/Chol to EryA, and CL to NudA [4,43,44].

We also found that, at pH 6.0, all four of the tested aegerolysins interact with an additional glycerophospholipid membrane receptor—phosphatidic acid (PA). For OlyA6, HetA, and Δ37VerA, this binding to PA dramatically decreased by increasing the pH. At pH 8.0, only NudA was able to interact with PA-supplemented lipid vesicles, exhibiting even stronger binding than at the lower pH values. PA is a simple glycerophospholipid which serves as a backbone for the synthesis of a number of classes of glycerophospholipids [45]. In biological samples, PA is estimated to account for 0.1–0.3 mol % of the total membrane lipids [46], where it is involved in in-receptor transport, exocytosis and phagocytosis, neuronal function, infectious diseases, and in cancer [45]. In 2015, a tetravalent peptide PAB-TP was shown to bind to as little as 1 mol % of PA in artificial lipid vesicles and was proposed as a new PA probe, but was not evaluated on the membranes of living cells [47]. The results of our binding studies indicate that Δ37VerA, which is the only thus far analyzed mushroom aegerolysin not interacting with membranes containing sphingolipids or other glycerophospholipids, could also be proposed as specific probe for labeling membrane PA at pH 6.0 in living or pre-fixed cells. 

The pH dependence of the affinity of different aegerolysins for lipid membranes observed in this study was already reported for ostreolysin, an aegerolysin from the mushroom *P. ostreatus*, which bears 78% amino acid identity with OlyA6 [48]. The binding of ostreolysin to sphingomyelin/cholesterol lipid vesicles was optimal in the pH range from 6.0 to 7.0, which is also the pH range in which this protein adopts a thermodynamically stable native-like conformation. This could explain, at least in part, the results obtained with NudA within this study, since the isoelectric points of both these proteins are around 5.0 [48, this study]. However, the isoelectric point of Δ37VerA, that shows a pH-dependent membrane activity similar to that of OlyA6 and NudA, dramatically differs from all of the other aegerolysins used in the study. It is thus tempting to speculate that rather than specific amino acids, or isoelectric points of individual proteins, the common protein fold of aegerolysins is important for the observed membrane interactions. Indeed, the models of the structures of aegerolysins investigated within this study at pH 7.0 indicate that all of the tested proteins exhibit a similar fold. So far, only the crystal structure of a complex of OlyA6 and SM exists [32]. Since the binding pocket of aegerolysins to which CPE, PA, or CL bind has not yet been determined, it is not possible to speculate which amino acid residues are necessary for binding to these lipids. 

We recently documented the membrane-permeabilizing activity of protein complexes in which the aegerolysin and the MACPF partners derive from different *Pleurotus* species [15]. In this study, we found that the HetA/PlyB combination is lytic for sphingolipid-containing vesicles, demonstrating that active pore-forming complexes could also be successfully formed by combining aegerolysin and MACPF protein partners from different mushroom genera. These combined results suggest that, in nature, two aegerolysin- and/or MACPF-protein producing fungi outside or within the same genus could cooperate in the same ecological niche. We were not able, however, to demonstrate the membrane-permeabilizing activity by also combining MucB with its non-native aegerolysin partners OlyA6, HetA, NudA, or Δ37VerA, probably because MucB bears an additional 15 amino acid residues which could affect the pore-formation or folding of the protein. In addition to HetA/PlyB, the permeabilization of sphingolipid-supplemented artificial membranes and erythrocytes was also confirmed for the NudA/NudB complexes but, similarly to the binding tests, their activity did not reach the potential of the OlyA6/PlyB protein complexes from *P. ostreatus*. In most of the cases, the membrane-permeabilizing activity increased with the lowering of the pH. At pH 6.0, the NudA/NudB complexes exhibited even higher effectivity in permeabilizing lipid vesicles reconstituted from the total insect cell lipids than the OlyA6/PlyB protein mixtures. This finding could propose the NudA/NudB complexes as new bioinsecticides for elimination of coleopteran pests that have pH-values around 6.0 in their midguts [49]. Indeed, all of the tested aegerolysin/MACPF complexes, including Δ37VerA/VerB, were found to be toxic for the Sf9 insect cells; OlyA6/PlyB again being the most effective. 

Interestingly, although some of the tested aegerolysins showed significant affinity for CL- and PA-containing vesicles in the binding tests, no lysis of these vesicles occurred when these aegerolysins were combined with MACPF protein partners. It seems that the lytic potential of mushroom aegerolysin/MACPF protein pairs is restricted to sphingolipid-containing membranes, possibly because of the lack of specific donor or acceptor groups of electrons in the glycerophospholipids in contrast to sphingolipids, or because the interaction of aegerolysins with glycerophospholipids involves different amino acid residues, which in turn hampers the interaction between aegerolysins and their MACPF protein partners. Furthermore, the stiffness and thickness of such membranes could also affect pore formation.

Although our study uncovers additional knowledge regarding mushroom aegerolysins deriving from species outside the *Pleurotus* genus, many questions still remain unanswered. According to our bioinformatic analysis, there are many Agaricomycetes coding only for aegerolysins, and not for their MACPF partner proteins. The biological role of these aegerolysins still remains enigmatic. Further, there are numerous Agaricomycetes which contain multiple (up to 24) aegerolysin homologues, and some of these mushrooms also code for numerous MACPF proteins. We speculate that selection pressure drove these mushrooms to express several cytolytic complexes, which in turn exhibit different effectivity and specificity towards individual membrane lipid species and consequently different toxicity towards specific taxa of competitors, pray, or enemies. In this regard, such aegerolysin-based cytolytic complexes could represent new lipid-sensing probes and potent biopesticides.

In comparison with previous studies on *Pleurotus* aegerolysins and aegerolysin/MACPF complexes [3,4,6,12,14,15,32,39,50], which were mostly performed at physiological pH values, this is also the first study highlighting the importance of pH for aegerolysin–membrane interactions. This finding should be considered when new mushroom aegerolysins are characterized, since our results clearly show that aegerolysins from different mushrooms can have quite different lipid-binding characteristics at different pH values. 

In conclusion, our results have broadened the spectrum of mushroom aegerolysin lipid receptors, showing that these proteins can effectively also bind selected glycerophospholipids, namely cardiolipin and the phosphatidic acid, in a pH-dependent manner. We also show that mushroom aegerolysins can form cytolytic complexes with their MACPF-partners only when their lipid receptor is sphingolipid and not glycerophospholipid. These findings could pave the way to further development of aegerolysin-based molecular probes for detecting cardiolipin or phosphatidic acid in cellular membranes.

## 4. Materials and Methods

### 4.1. Reagents and Materials

#### 4.1.1. Enzymes, Kits, Chemicals, and Cells

The FastDigest restriction enzymes *Nde*I, *Xho*I, *Bam*HI, *Mlu*I, rapid DNA ligation kit, GeneJET PCR purification kit, GeneJET gel extraction kit, GeneJET plasmid miniprep kit, PageRuler prestained protein ladder, and 1 kb Plus DNA ladder were all from Fermentas (Thermo Fisher Scientific, Waltham, MA, USA). Benzoase, RNAse, protease inhibitors, and pET plasmids were from Novagen (Merck, Darmstadt, Germany). Insect cells derived from the ovarian epithelial cells of the fall army worm (*Spodoptera frugiperda*; Sf9 cells; Thermo Fisher Scientific, Waltham, MA, USA) were maintained in a continuous suspension culture under serum-free conditions at 28 °C in Insect XPRESS protein-free insect cell medium with l-glutamine (Lonza, Basel, Switzerland), with agitation at 150 rpm.

#### 4.1.2. Genes, Primers, and Plasmids

Genes coding for nudolysin A (NudA; NCBI Reference Sequence: KAF9458124.1) and nudolysin B (NudB; NCBI Reference Sequence: KAF9458125.1) from *L. nuda*, mucolysin A (MucA; NCBI Reference Sequence: KAF8919893.1), and mucolysin B (MucB; NCBI Reference Sequence: KAF8919894.1) from *M. mucida*, heterolysin A (HetA; NCBI Reference Sequence: XP_009545192.1) and heterolysin B (HetB; NCBI Reference Sequence: XP_009544624.1) from *H. irregulare* TC 32-1 were optimized for expression in *Escerichia coli* and synthesized by Kemomed d.o.o. as linear DNA products. Genes coding for versicolysin A (VerA; NCBI Reference Sequence: XP_008043601.1) and versicolysin B (VerB; NCBI Reference Sequence: XP_008043602.1) from *T. versicolor* FP-101664 SS1 were optimized for expression in *E. coli* and synthesized by Genescript (Piscataway, NJ, USA). Gene *verA* was purchased as already inserted in *E. coli* expression vector pET21b(+), while *verB* was purchased as inserted in *E. coli* expression vector pET28a(+). The N-terminus deletion mutant of *verA* (Δ37*verA*) was created using a PCR reaction. Primers for amplification of *nudA*, *nudB*, *mucA*, *mucB*, *hetA*, *hetB*, and Δ37*verA* were synthesized by Microsynth (AUT) (Table S5). Aegerolysins (NudA, HetA, MucA, VerA, and Δ37VerA) were prepared as C-terminally His-tagged proteins, while MACPF domain-containing proteins partners (NudB, HetB, MucB, and VerB) were prepared as N-terminally His-tagged proteins in appropriate plasmid vectors (Table S6).

#### 4.1.3. Lipids

Cholesterol (wool-derived, ovine Chol), sphingomyelin (brain, porcine SM), ceramide phosphoethanolamine (brain, porcine CPE), cardiolipin (heart, bovine CA), L-α-phosphatidylethanolamine (egg PE), L-α-phosphatidylserine (brain, PS), 1,2-dioleoyl-sn-glycero-3-phosphate (18:1 PA), and 1-palmitoyl-2-oleoyl-sn-glycero-3-phosphocholine (POPC) were from Avanti Polar Lipids (Alabaster, AL, USA). Total lipids from the bovine red blood cells (RBC) and Sf9 insect cell line were extracted according to Bligh and Dyer [51], and stored under liquid nitrogen at −80 °C.

### 4.2. Bioinformatics

#### 4.2.1. Identification of Aegerolysin-Coding Basidiomycetes

To identify aegerolysins in the class Agaricomycetes, the fungal genome database JGI MycoCosm [23,24] was searched for the Pfam protein domain PF06355. Identified aegerolysin-containing species were taxonomically classified according to the NCBI Taxonomy website [25]. To identify MACPF proteins in these fungal species, the same genome database JGI was searched for the Pfam protein domain PF01823. In genomes with aegerolysin singlets, an additional search was performed using JGI Blast with sequence of OlyA6 and PlyB (E 1.0 × 10^−5^). The Aegerolysin and MACPF gene pairs were identified by manual examination of appropriate contigs.

#### 4.2.2. Phylogenetic Analysis

Phylogenetic analysis of muscle-aligned aegerolysins and inferred by the maximum likelihood method was performed using Molecular Evolutionary Genetics Analysis, version 11 (MEGA11) [26,27]. The identified aegerolysin-containing species were taxonomically classified according to the NCBI Taxonomy website [25].

#### 4.2.3. Calculated Basic Biochemical Characteristic of Aegerolysins and Their MACPF Domain-Containing Protein Partners

The basic biochemical properties (molecular weights and isoelectric points) of aegerolysins and their MACPF-domain protein partners were calculated for native proteins and for their recombinant variants using ExPASy ProtParm tool (https://web.expasy.org/protparam/; 10 December 2022). Pairwise protein identities for aegerolysins and their MACPF-domain protein partners were calculated using Basic Local Alignment Search Tool (BLAST) (https://blast.ncbi.nlm.nih.gov/Blast.cgi; 10 December 2022).

#### 4.2.4. Sequence Alignment, Prediction of Protein Secondary Structures, Signal Peptides, and Protein Localisation Prediction

A multiple sequence alignment was performed with the workbench editor Jalview version 2.11.2.7 using the muscle algorithm [52]. The protein secondary structure prediction server JPred4, accessible through Jalview, was used to predict the secondary structure of proteins [53]. The SignalP 6.0 algorithm predicts signal peptides and the location of their cleavage sites using a machine learning model in proteins from Eukarya [37]. As the prediction program for the subcellular localization DeepLoc 2.0 was used [38].

#### 4.2.5. Protein Model Generation

The deep learning algorithm AlphaFold2 was used to model the protein structure [30]. UCSF ChimeraX version 1.5 (8 December 2022) and UCSF ChimeraX version 1.4 (24 November 2022) were used to run AlphaFold2 in conjunction with a free and accessible platform for protein folding ColabFold [34,54]. 

#### 4.2.6. Protein Structure Visualization

Cartoon representation of the structures and models was performed using PyMOL, version 2.2.0 [31]. In protein crystallography, the B-factor (the Debye-Waller factor, the temperature factor, or the atomic displacement parameter) describes the attenuation of X-ray or neutron scattering by thermal motion. In the models, the lDDT, a superposition-free score, evaluates the local distance differences of all atoms, including the validation of stereochemical plausibility. The protein molecules were colored according to the Eisenberg hydrophobicity scale [35]. Electrostatic potentials at pH 7.0 were calculated from negative (red) to positive (blue) (scale ranges from +/−5) using the adaptive Poisson–Boltzmann solver (APBS) module integrated in PyMOL [36]. 

### 4.3. Preparation of Recombinant Proteins

The recombinant proteins OlyA6, NudA, VerA, Δ37VerA, MucA, HetA, and Δ48PlyB (henceforth PlyB), Δ21HetB (henceforth HetB), Δ29VerB (henceforth VerB), Δ30NudB (henceforth NudB), and Δ43NudB (henceforth NudB) were produced as described previously [3,12], but with minor corrections as described in Supplementary Materials and Methods.

### 4.4. Preparation of Artificial Lipid Vesicles

#### 4.4.1. Preparation of Lipid Mixtures and Multilamellar Vesicles

Mixtures of natural and synthetic lipids were used to determine lipid receptors of aegerolysins. We prepared two natural lipid mixtures from the total lipid extract from RBC, representing the mammalian lipid composition, and from the insect cell line Sf9, representing the invertebrate lipid composition. Additionally, seven different lipid mixtures were prepared from commercially available lipids. These were first accurately weighed on an analytical scale and then dissolved in chloroform, except for CPE, which was dissolved in a mixture of chloroform and methanol (9:1, V:V). In pre-washed flasks, volumes of the dissolved lipids were pipetted off to obtain mixtures consisting of lipids in equimolar lipid composition with the formula X:POPC:Chol, where the lipid X was either CPE, PS, PE, CL, or PA. Alternatively, equimolar lipid compositions with the formula X:Chol, where the lipid X was either SM or POPC, were prepared. The POPC:Chol lipid mixture was used as a negative control. After the preparation of lipid films using a rotary evaporator, and after removing the organic solvent, each natural and artificial lipid mixture was swollen in three different vesicle buffers composed of 20 mM HEPES, 140 mM NaCl (pH 7.0 or 8.0) or 20 mM MES, 140 mM NaCl (pH 6.0), at room temperature, to a final lipid concentration of 5 mg/mL, and vortexed vigorously to give multilamellar vesicles (MLVs). The prepared vesicles were stored at −20 °C.

#### 4.4.2. Preparation of Large Unilamellar Lipid Vesicles

Large unilamellar vesicles (LUVs) with a diameter of ~100 nm were prepared from MLVs using an extrusion through a 100 nm polycarbonate filter (Avestin, Ottawa, ON, Canada) mounted in a small-volume extruder (Avestin, Ottawa, ON, Canada), at a temperature of ~60 °C as described previously [55]. The LUVs were prepared in the final concentration of 1 mg/mL in vesicle buffer (pH 6.0, 7.0, or 8.0).

#### 4.4.3. Preparation of Small Unilamellar Vesicles with Calcein

First, MLVs were prepared by the same procedure as described above (Section 2.4.1), except that 0.5 mL of calcein buffer (800 μL 10 M NaOH, 1.24 g calcein, water up to 25 mL) was added to the lipid mixtures instead of vesicle buffers and vortexed vigorously. The obtained MLVs were sonicated for 20 min at an amplitude of 40%, with an interval of 10 s pulse and 10 s pause, to give small unilamellar vesicles (SUVs). Extra-vesicular calcein was removed by gel filtration on a SephadexG-50 column, which was eluted with vesicle buffer. These SUVs were stored at 4 °C and used within 2 days.

### 4.5. Sedimentation Assay

MLVs (5 mg/mL; prepared in vesicle buffer, pH 8.0) were combined with 1 μg of each of the aegerolysins (OlyA6, NudA, HetA, and Δ37VerA), to obtain the 1:3000 molar protein/lipid ratio. For each aegerolysin, a control experiment without MLVs was performed to evaluate eventual protein spontaneous aggregation during sedimentation. The mixtures were incubated for 30 min while shaking at 600 rpm. After incubation, the aegerolysin/MLV mixtures were centrifuged at 16.100× *g* for 30 min at 25 °C. The supernatants were transferred to new Eppendorf tubes and the sediments were resuspended in 100 μL vesicle buffer (pH 8.0) and centrifuged again under the same conditions for 20 min. The supernatants were then discarded, and the sediments were resuspended in 16 μL of dH2O and separated by SDS-PAGE. Analysis of the SDS-PAGE gel was performed using the GelQuantNET program, which allowed the quantification of the intensity of the protein spots on the gels after electrophoresis. In this way, we estimated the percentage of protein binding to MLVs.

### 4.6. Surface Plasmon Resonance Measurements

Using the surface plasmon resonance (SPR) method, we measured the interaction of each aegerolysin (OlyA6, NudA, HetA, and Δ37VerA; 5 µM) with nine types of LUVs, representing natural and artificial lipid mixtures, at three pH values (6.0, 7.0, and 8.0). For the interaction measurements, we used a refractometer Biacore T200, L1 sensor chip, and the BIA evaluation software (version 3.2.1) package for data processing. The LUVs were bound as ligands to the L1 sensor chip. At the beginning of the experiment, we equilibrated the L1 sensor chip in a time interval of 30 min at 25 °C. To prevent nonspecific binding of the analyzed proteins, we used 0.1 mg/mL of BSA, and the solution was applied at a flow rate of 30 µL/min. Measurements of protein interactions with lipid membranes were performed on Flowcell 4 (4Fc4), to which we applied the prepared lipid vesicles at a time interval of 10 min at a flow rate of 2 µL/min. Fc3 was used as a reference cell to which BSA was applied. The proteins were injected at a flow rate of 10 µL/min and allowed to associate for 1 min. Regeneration of the chip was performed with 0.5% SDS, 40 mM of octyl-beta-glucoside at a flow rate of 10 µL/min and 30% ethanol. Interaction metrics were performed at 25 °C. 

The stability of the vesicles and the quantity of immobilized vesicles are pH-dependent processes. Since these two factors influence the quantity of bound proteins, we have normalized the response in response units (RUs) for each aegerolysin at 100 s with the quantity of immobilized vesicles. The normalized factors we obtained revealed at which pH the binding of aegerolysins for each of the nine lipid mixtures is the most efficient. 

### 4.7. Calcein Release Assay

SUVs loaded with calcein at the self-quenching concentration (80 mM) were prepared as described previously [55]. Vesicle permeabilization was monitored using a fluorescence microplate reader (Anthos, UK) with excitation and emission set at 485 nm and 535 nm, respectively. Calcein-loaded vesicles were exposed to mixtures of aegerolysins (OlyA6, NudA, HetA, or Δ37VerA) which showed some extent of binding to LUVs, and their respective native MACPF protein partners (PlyB, NudB, or VerB) at a molar ratio of 10:1. The protein mixtures were tested in a concentration range from 5 to 0.005 μM. In the case of HetA, the combination of HetA/PlyB was used since we did not obtain recombinant HetB. The experiments were run for 20 min at 25 °C. The permeabilization induced by the mixtures of aegerolysin and their native MACPF protein partners was expressed as the percentage of the maximal permeabilization obtained after the addition of the detergent Triton-X 100 to a final concentration of 1 mM.

### 4.8. Hemolytic Assay

The hemolytic activities of the protein mixtures (OlyA6/PlyB, NudA/NudB, or Δ37VerA/VerB) were measured on a kinetic microplate reader (Tecan, Switzerland) at ∼25 °C, as described previously [56], at different pH values (6.0, 7.0, and 8.0). The RBCs were first washed twice with an erythrocyte buffer (140 mM NaCl, 20 mM Tris-HCl, pH 7.4) and then twice with a buffer with the desired pH value (20 mM MES, 140 mM NaCl, pH 6.0 or 20 mM TRIS, 140 mM NaCl, pH 7.0 or 8.0). The final density of the RBC suspension at the desired pH value was adjusted to an apparent absorbance of 1.0 at 630 nm (A_630_). The Aegerolysins (OlyA6, NudA, and Δ37VerA) and their native MACPF partner proteins (PlyB, NudB, and VerB) were mixed in the microtiter plates in a 10:1 molar ratio in the final aegerolysin concentrations of 1, 0.1, and 0.01 µM. In the case of HetA, the combination of HetA/PlyB was used since we did not obtain recombinant HetB. As a control, a 1 µM concentration of each aegerolysin or a 0.1 µM concentration of each MACPF protein alone was tested at all three pH values. Then, the volumes in the wells were increased to 100 µL by the addition of the buffer with appropriate pH values. An amount of 100 µL of the previously prepared RBC suspension was added to each well using a multichannel pipette, and the turbidity of the RBC suspension was monitored at a wavelength of 630 nm for 1 h. 

### 4.9. Sf9 Cytotoxic Activity

The cytotoxicity of the aegerolysin/MACPF protein mixtures was measured using the colorimetric MTT Assay (Thermo Fisher Scientific, Waltham, MA, USA) that uses MTT (3-(4,5-dimethylthiazol-2-yl)-2,5-diphenyltetrazolium bromide). Insect Sf9 cells were plated in 96-well microtiter plates (Brand, Wertheim, Germany) at a density of 2.5 × 10^5^ cells/well in a growth medium (ESF 921, Expression Systems, Davis, CA, USA) with a pH value of 6.3 at 28 °C in a humidified chamber. After 48 h, the cell medium was removed and the protein mixtures (OlyA6/PlyB, NudA/NudB, HetA/PlyB, and Δ37VerA/VerB) dissolved in the culture medium were added to the final volume of 100 μL. The molar ratios of aegerolysins and their native MACPF protein partners were set to 10:1, and the final concentrations of aegerolysins were 5, 1, 0.5, 0.1, and 0.5 µM. The cells overlayed with the protein mixtures were incubated for 1 h at 28 °C. After incubation, the proteins mixtures were removed, MTT was added, and the cells were incubated for an additional 3 h at 28 °C. Then, the MTT solution was removed and DMSO was added and incubated with the cells for 30 min at room temperature. Finally, we measured the absorbance at different wavelengths (570 and 630 nm) using an EL-800 microplate reader (Tecan, Männedorf, Switzerland). The measurement, at a wavelength of 630 nm, served as the background of the measurement, which was then subtracted from the measurement at 570 nm. The cytotoxicity test for each cytotoxic sample and for their corresponding dilutions was repeated 3 times.

## Figures and Tables

**Table 1:** The hemolytic activity of the OlyA6/PlyB, NudA/NudB, Δ37VerA/VerB, and HetA/PlyB protein pairs at pH values of 6.0, 7.0, and 8.0. The time course of 100% hemolysis was monitored with a microplate reader at 630 nm. The molar ratio between aegerolysin and their native MACPF protein partners was set at 10:1.

Protein Mixture (10:1)	Concertation of an Aegerolysin in the Protein Mixture	pH 6.0	pH 7.0	pH 8.0
		Time of Hemolysis (min)	Time of Hemolysis (min)	Time of Hemolysis (min)
OlyA6/PlyB	1 µM of OlyA6	0.7 ± 0.0	0.7 ± 0.0	0.7 ± 0.0
	0.1 µM of OlyA6	0.7 ± 0.0	4 ± 1.1	2 ± 0.0
	0.01 µM of OlyA6	2.7 ± 0.5	20.6 ± 2.1	26.4 ± 1.4
NudA/NudB	1 µM of NudA	0.7 ± 0.0	0.7 ± 0.0	2 ± 0.0
	0.1 µM of NudA	1.3 ± 0.4	11.1 ± 1.1	/
	0.01 µM of NudA	48.8 ± 3.9	/	/
Δ37VerA/VerB	1 µM of Δ37VerA	/	/	/
	0.1 µM of Δ37VerA	/	/	/
	0.01 µM of Δ37VerA	/	/	/
HetA/PlyB	1 µM of HetA	0.7 ± 0.0	1.3 ± 0.3	0.7 ± 0.0
	0.1 µM of HetA	0.7 ± 0.0	8.2 ± 1.0	5.3 ± 0.9
	0.01 µM of HetA	9.8 ± 1.2	/	/

OlyA6, ostreolysin A6; PlyB, pleurotolysin B; NudA, nudolysin A; NudB, nudolysin B; Δ37VerA, deletion mutant of versicolysin A; VerB, versicolysin B HetA, heterolysin A; /, 100% hemolysis did not occur. The mean values of triplicates with standard errors are shown.

## Data Availability

The original contributions presented in the study are included in the article/Supplementary Material, further inquiries can be directed to the corresponding author.

## Supplementary Materials

The following supporting information can be downloaded at: https://www.mdpi.com/article/10.3390/toxins16030143/s1, Table S1: Aegerolysins from the class of Agaricomycetes in species with published genomes [57,58,59,60,61,62,63,64,65,66,67,68,69,70,71,72,73,74,75,76,77,78]; Table S2: Basic biochemical characteristics of native aegerolysins analyzed in the study; Table S3: Basic biochemical characteristics of native MACPF proteins analyzed in the study; Table S4: The amounts of immobilized vesicles in RUs for each SPR experiment; Table S5: The list of primers used in the study; Table S6: The list of proteins, corresponding vectors, cloning sights, and sizes of expressed proteins; Table S7: Signal peptide prediction; Table S8: Protein localization prediction; Figure S1: Phylogenetic analysis of aegerolysins from Agaricomycetes [79]; Figure S2: Aegerolysin gene loci in fungal genomes; Figure S3: Alignments of amino acid sequences and prediction of secondary structures of selected aegerolysins and MACPF proteins; Figure S4: Assessment of OlyA6, HetA, NudA, and Δ37VerA association with lipid vesicles at pH 8.0; Figure S5: SPR analysis of interaction of aegerolysins with LUVs containing glicerophospholipid receptors; Figure S6: Hemolytic activity of 1 µM of aegerolysins or 0.1 µM of MACPF proteins at pH 6.0, 7.0, and 8.0; Figure S7: Effect of aegerolysins and their native MACPF protein partners alone on survival of Sf9 cells.
