# Supplementary material for: New Insights into Interactions between Mushroom Aegerolysins and Membrane Lipids
Source: Toxins (Basel). 2024 Mar 9;16(3):143. doi: 10.3390/toxins16030143 (PMC10975569; doi:10.3390/toxins16030143)
Supplement: Supplementary file 1 [file toxins-16-00143-s001.zip › toxins-2874105-supplementary.pdf]

Supplementary Materials: New Insights into Interactions between Mushroom Aegerolysins and Membrane Lipids

Larisa Lara Popošek, Nada Kraševec, Gregor Bajc, Urška Glavač, Matija Hrovatin, Žan Perko, Ana Slavič, Miha

1. Supplementary Materials and Methods

1.1. Expression and Purification of Aegerolysins and their MACPF Protein Partners

The aegerolysins OlyA6, NudA, HetA, VerA, Δ37VerA, and MucA were constructed as C-terminally tagged His_6 (H_6) fusion proteins. These proteins were expressed as soluble proteins in the BL21(DE3) or in the BL21(DE3) pLysE *E. coli* strains. Their MACPF protein partners Δ48PlyB (henceforth PlyB), Δ21HetB (henceforth HetB), Δ29VerB (henceforth VerB), Δ30NudB (henceforth NudB) and Δ43NudB (henceforth NudB) were constructed as N-terminally tagged His_6 (H_6) fusion proteins and expressed in inclusion bodies in the BL21(DE3) *E. coli* strains and then refolded. After transformation, the cells were grown on LB plates with 100 μg/mL of ampicillin (Sigma, USA) (LBAp) or with 50 μg/mL of kanamycin (Sigma, USA) (LBKn). A single colony was then inoculated into 100 mL LBAp or LBKn medium, and grown overnight at 37 °C on a rotating wheel. The overnight culture (5 mL) was inoculated into 500 mL LBAp or LBKn medium. Typically, 1 L of bacterial culture were grown at 37 °C up to an optical density (A_{600}) of 0.6 to 0.8. After induction of the production of the recombinant proteins using 0.5 mM isopropyl β-D-1-thiogalactopyranoside (IPTG) (Sigma, USA), bacterial growth was continued overnight at 22 °C. The bacteria were then centrifuged for 30 min at 4.370×*g* and 4 °C. The bacterial pellet was kept on ice during homogenization. The cells were resuspended at (2 ml buffer/g dry weight bacteria) in lysis buffer (50 mM NaH_2PO_4, 300 mM NaCl, 10 mM imidazole, pH 8.0), supplemented with lysozyme (0.5 mg/mL) (Merck, USA), benzoase (5 U/mL) (Merck, USA), RNAse (20 μg/mL) (Sigma, USA), protease inhibitors (1 tablet/10 mL) (Roche, Switzerland), and β-mercaptoethanol (20 mM) (Sigma, USA), shaken for 30 min at 4 °C, and sonicated (Vibracell, Sonics, USA) at 40 % amplitude for 10 min on ice. The homogenate was centrifuged for 30 min at 26.323×*g* at 4 °C.

1.1.1. Isolation of recombinant aegerolysins OlyA6, NudA, HetA, VerA, Δ37VerA, and MucA

After centrifugation the obtained supernatants were kept at 4 °C, and the pellets were resuspended again in lysis buffer (1 ml buffer/g dry weight bacteria). Shaking for 30 min on ice was followed by 10 min of sonication on ice and centrifugation (30 min at 26.323×*g* at 4 °C). Both supernatants were then merged and filtered through a 0.2-μm cellulose-acetate filter and then loaded onto a 1.5 mL Ni^{2+}-NTA column equilibrated in buffer A containing 50 mM NaH_2PO_4, 300 mM NaCl, 10 mM imidazole and 20 mM β-mercaptoethanol, pH 8.0. Non-specifically bound proteins were eluted with 50 mM of NaH_2PO_4, 300 mM of NaCl, 20 mM of imidazole, pH 8.0, and the residual proteins were eluted with 300 mM of imidazole in the same buffer. After elution, the sample buffer was exchanged for 20 mM of HEPES, 140 mM of NaCl, pH 8.0, and was used in the further purification on size exclusion column (GE Healthcare, UK). After elution, the sample buffer was exchanged again for 20 mM of HEPES, 140 mM of NaCl (pH 7.0 or 8.0), or 20 mM of MES 140 mM of NaCl (pH 6.0).

1.1.2. Isolation of recombinant MACPF proteins partners PlyB, HetB, NudB, MucB, and VerB

To refold insoluble MACPF proteins PlyB, NudB, HetB, VerB, and MucB the pellets after centrifugation were resuspended in 20 mM Tris-HCl, pH 8.0 (3 mL buffer/g wet mass), sonicated for 10 min on ice (40 % amplitude) and then centrifuged for 30 min at 26.323×g. The inclusion bodies were then washed in 20 mM Tris-HCl, 0.5 M NaCl, 2 M

urea, 2 % (v/v) Triton X-100, pH 8.0 (5 mL/g of sediment wet mass). Sonication and centrifugation were repeated, and the pellets were resuspended in the same buffer without urea and centrifuged again, as before. The washed inclusion bodies were then dissolved by stirring the inclusion bodies overnight at 4 ºC in 20 mM of Tris-HCl, 0.5 M of NaCl, 6 M of guanidinium chloride, 5 mM of imidazole, 5 mM of β-mercaptoethanol, at pH 8.0 (3 mL of buffer/g inclusion body wet mass), and centrifuged for 30 min at 17.000×g at 4 ºC. The dissolved proteins were refolded by 100-fold dilution in 20 mM of Tris-HCl, 0.5 mM of NaCl, 5 mM of imidazole, 5 mM of β-mercaptoethanol, at pH 8.0 and at 4 ºC. The insoluble materials were removed by 30 min centrifugation at 17.000×g, and the refolded proteins were purified on a Ni^{2+}-NTA column. After elution, the sample buffer was exchanged for 140 mM of NaCl, 20 mM of Tris-HCl, and 5 % (v/v) glycerol at pH 8.0 and stored at -20 °C. Freshly thawed aliquots were used in the functional studies, to minimize spontaneous protein degradation.

2. *Supplementary tables*

Table S1. Aegerolysins from the class Agaricomycetes in species with published genomes.

Genome	Order	Aegerolysin PF06355	OlyA6 JGI Blast	Aegerolysin Protein Number	MACPF PF01823	PlyB JGI Blast	Genome Reference
Galerina marginata v1.0	Agaricales	1	0	Galma1|143730	0	0	[57]
Lepista nuda CBS 247.69 v1.0	Agaricales	1	1	Lepnud1|1174238*	0	1	[58]
Oudemansiella mucida CBS 558.79 v1.0 (*Mucidula mucida*)	Agaricales	1	1	Oudmuc1|1429373*	0	1	[58]
Pleurotus ostreatus PC15 v2.0	Agaricales	1	1	Pleo-sPC15_2|1090164*[,s]	2	1[#]	[57]
Pleurotus ostreatus PC9 v1.0	Agaricales	1	1	PleosPC9_1|72745*	3	1[#]	[59]
Pterula gracilis CBS309.79 v1.0	Agaricales	1	0	Ptegra1|557421[c,s]	0	0	[60]
Gyrodon lividus BX v1.0	Boletales	1	0	Gyrli1|877364	0	0	[61]
Paxillus ammoniavirescens Pou09.2 v1.0	Boletales	1	0	Paxam1|1014777	0	0	[61]
Paxillus adelphus Ve08.2h10 v2.0	Boletales	1	0	Paxru2|1809802	0	0	[62]
Pisolithus croceorrhizus subspA 74A v1.0	Boletales	1	0	Piscro1|383570	1	0	[63]
Pisolithus sp. B1 v1.0	Boletales	1	0	Pismi_AB1_1|1098592	1	0	[63]
Pisolithus orientalis OTSU v2.0	Boletales	1	1	Pisori2|840897	0	1	[63]
Pisolithus thermaeus 11 v1.0	Boletales	1	0	Pisthe1|1755645	1	1	[63]
Pisolithus croceorrhizus ssp. 2 72 v1.0	Boletales	1	0	Pisthe227_1|2266095	0	1	[63]
Serpula himantioides (*S. lacrymans* var *shastensis*) MUCL38935 v1.0	Boletales	1	1	Serla_varsha1|57805[c,s]	0	0	[64]
Punctularia strigosozonata v1.0	Corticiales	1	1	Punst1|101965	0	0	[65]
Cytidiella melzeri FP 102339 v1.0	Polyporales	1	1	Cytmel1|219606	25	0	[66]
Dichomitus squalens LYAD-421 SS1 v1.0	Polyporales	1	1	Dicsq1|69680	0	0	[65]
Dichomitus squalens OM18370.1 v1.0	Polyporales	1	1	Dicsqu18370_1|667277[c,s]	0	0	[67]
Dichomitus squalens CBS463.89 v1.0	Polyporales	1	1	Dicsqu463_1|869446[c,s]	0	0	[67]
Dichomitus squalens CBS464.89 v1.0	Polyporales	1	1	Dicsqu464_1|982219	0	0	[67]
Hydnopolyporus fimbriatus CBS384.51 v1.0	Polyporales	1	0	Hydpi2|169438	0	2	[66]
Irpex lacteus CCBAS Fr. 238 617/93 v1.0	Polyporales	1	0	Irplac1|1724138[c,s]	5	0	[66]
Pycnoporus puniceus CIRM-BRFM 1868 v1.0	Polyporales	1	1	Pycpun1|203258*	1	1	[61]
Trametes betulina CIRM-BRFM 1801 v1.0	Polyporales	1	1	Trabet1|845770*	1	1	[66]
Trametopsis cervina CIRM-BRFM 1824 v1.0	Polyporales	1	1	Trace1|560779	4	0	[66]
Trametes gibbosa CIRM-BRFM 1770 v1.0	Polyporales	1	1	Tragib1|1322232[c,s]	0	1	[66]
Trametes maxima CIRM-BRFM 1813 v1.0	Polyporales	1	1	Tramax1|336724	1	0	[66]

Genome	Order	Aegerolysin PF06355	OlyA6 JGI Blast	Aegerolysin Protein Number	MACPF PF01823	PlyB JGI Blast	Genome Reference
Trametes meyenii CIRM-BRFM 1810 v1.0	Polyporales	1	1	Tramey1|1013932	0	0	[66]
Trametes pubescens FBCC735	Polyporales	1	1	Trapub1|5034	0	2	[68]
Trametes versicolor v1.0	Polyporales	1	1	Trave1|52920*	0	1[#]	[65]
Heterobasidion annosum v2.0	Russulales	1	1	Hetan2|148469*	0	1[#]	[69]
Crepidotus variabilis CBS 506.95 v1.0	Agaricales	2	-	Crevar1|769707<br>Crevar1|838031	-	-	[58]
Hypholoma sublateritium v1.0	Agaricales	2	-	Hypsu1|208850<br>Hypsu1|208851	-	-	[62]
Laccaria amethystina LaAM-08-1 v2.0	Agaricales	2	-	Lacam2|687022<br>Lacam2|1678639	-	-	[62]
Moniliophthora roreri MCA 2997	Agaricales	2	-	MroMCA2997_1|8059<br>MroMCA2997_1|12739[c]	-	-	[70]
Pleurotus eryngii ATCC 90797 v1.0	Agaricales	2	-	Pleery1|1387261<br>Pleery1|1444518	-	-	[58]
Paxillus involutus ATCC 200175 v1.0	Boletales	2	-	Paxin1|9942<br>Paxin1|9943	-	-	[62]
Pisolithus albus SI12 v1.0	Boletales	2	-	Pisalb1|1121001<br>Pisalb1|2167476	-	-	[63]
Pisolithus microcarpus 441 v2.0	Boletales	2	-	Pismi2|4275208<br>Pismi2|688450	-	-	[62]
Fibroporia radiculosa TFFH 294	Polyporales	2	-	Fibra1|8583<br>Fibra1|8593			[71]
Lyophyllum atratum CBS 144462 v1.0	Agaricales	3	-	Lyoat1|1430114<br>Lyoat1|1453442<br>Lyoat1|1456029	-	-	[72]
Volvariella volvacea V23	Agaricales	3	-	Volvo1|116806<br>Volvo1|121057<br>Volvo1|121539	-	-	[73]
Pisolithus marmoratus ssp. 2 16A v1.0	Boletales	3	-	Pismar1|2600390<br>Pismar1|2666719<br>Pismar1|2685475	-	-	[63]
Lentinus tigrinus ALCF2SS1-6 v1.0	Polyporales	3	-	Lenti6_1|580338<br>Lenti6_1|580340<br>Lenti6_1|580351	-	-	[74]
Lentinus tigrinus ALCF2SS1-7 v1.0	Polyporales	3	-	Lenti7_1|523664<br>Lenti7_1|523675<br>Lenti7_1|571802	-	-	[74]
Polyporus squamosus CCBS 676 v1.0	Polyporales	3	-	Polsqu1573877<br>Polsqu1645443<br>Polsqu1707804	-	-	[66]
Lactifluus cf. subvellereus BPL653 v1.0	Russulales	3	-	Lacsub1|1211756<br>Lacsub1|1211789<br>Lacsub1|1211835	-	-	[75]
Lanmaoa asiatica v1.0	Boletales	4	-	Lanmao1|5926[c]<br>Lanmao1|7528<br>Lanmao1|9032<br>Lanmao1|9180	-	-	[76]
Phlebopus sp. FC_14 v2.0	Boletales	4	-	PhlFC14_2|483767<br>PhlFC14_2|1801769<br>PhlFC14_2|1827079<br>PhlFC14_2|1947475	-	-	[76]
Sphaerobolus stellatus v1.0	Geastrales	4	-	Sphst1|29870<br>Sphst1|164791<br>Sphst1|191975<br>Sphst1|267302	-	-	[62]
Obba rivulosa 3A-2 v1.0	Polyporales	4	-	Obbri1|189930<br>Obbri1|724008[c]<br>Obbri1|862642<br>Obbri1|887022[c]	-	-	[77]
Polyporus brumalis CIRM-BRFM 1820 v1.0	Polyporales	4	-	Polbr1|493721<br>Polbr1|1403104<br>Polbr1|1403107<br>Polbr1|1482675[c]	-	-	[61]
Russula rugulosa BPL654 v1.0	Russulales	4	-	Rusrug1|922004	-	-	[75]

Genome	Order	Aegerolysin PF06355	OlyA6 JGI Blast	Aegerolysin Protein Number	MACPF PF01823	PlyB JGI Blast	Genome Reference
				Rusrug1|925929<br>Rusrug1|1001040<br>Rusrug1|1191120			
Moniliophthora perniciosa FA553	Agaricales	6	-	Monpe1_1|83513<br>Monpe1_1|83795<br>Monpe1_1|84536[c]<br>Monpe1_1|86512<br>Monpe1_1|91804<br>Monpe1_1|92526	-	-	[78]
Polyporus arcularius v1.0	Polyporales	6	-	Polar1|492019<br>Polar1|547014<br>Polar1|586868<br>Polar1|591849<br>Polar1|598570<br>Polar1|606953	-	-	[60]
Amanita rubescens Přilba v1.0	Agaricales	7	-	Amarub1|650716<br>Amarub1|970520<br>Amarub1|991530<br>Amarub1|1079426[c]<br>Amarub1|1082242[c]<br>Amarub1|1111223[c]<br>Amarub1|1161814	-	-	[61]
Panaeolus papilionaceus CIRM-BRFM 715 v1.0	Agaricales	7	-	Panpap1|19875<br>Panpap1|689993<br>Panpap1|1355982<br>Panpap1|1397581<br>Panpap1|1533752<br>Panpap1|1610024<br>Panpap1|1614630[c]	-	-	[58]
Leucogyrophana mollusca KUC20120723A-06 v1.0	Boletales	15	-	Leumo1|1006919[c]<br>Leumo1|1015671<br>Leumo1|1031743<br>Leumo1|1031757<br>Leumo1|1040248<br>Leumo1|1041010<br>Leumo1|1041657<br>Leumo1|1042072<br>Leumo1|1050645<br>Leumo1|1097675<br>Leumo1|1099592<br>Leumo1|1130958<br>Leumo1|1133906<br>Leumo1|1133910<br>Leumo1|1134698	-	-	[76]
Hygrophoropsis aurantiaca ATCC 28755 v1.0	Boletales	24	-	Hygaur1|163564<br>Hygaur1|720682<br>Hygaur1|1017232[c]<br>Hygaur1|1017358<br>Hygaur1|1102255<br>Hygaur1|1106052<br>Hygaur1|1157025<br>Hygaur1|1161011<br>Hygaur1|1164082<br>Hygaur1|1164280<br>Hygaur1|1164288<br>Hygaur1|1164298[c]<br>Hygaur1|1164886<br>Hygaur1|1164907<br>Hygaur1|1165900[c]<br>Hygaur1|1166697<br>Hygaur1|1166711<br>Hygaur1|1166992<br>Hygaur1|1167771<br>Hygaur1|1167895<br>Hygaur1|1168331<br>Hygaur1|1168496<br>Hygaur1|1168858<br>Hygaur1|1168971[c]	-	-	[78]

Aegerolysins (160) from published fungal genomes (60) identified in the JGI MycoCosm database https://doi.org/10.1007/978-1-0716-2871-3_14; *, Aegerolysin and MACPF gene pair; [c], omitted from aegerolysin phylogenetic tree; [s], omitted from phylogenetic tree out inferred from of singlet aegerolysins; [#], manual annotation; MACPF, membrane attack complex/perforin.

Table S2. Basic biochemical characteristics of native aegerolysins analyzed in the study.

	Number of amino acids	MW (kDa)	Ip	% Amino acid identity with OlyA6
OlyA6	138	15.1	5.5	100
NudA	138	15.2	5.3	81.9
MucA	121	13.3	6.9	75
HetA	137	15.0	6.7	73.9
VerA	174	19.2	5.1	55.1
Δ37VerA	138	15.2	7.7	55.1

OlyA6, ostreolysin A6; NudA, nudolysin A; MucA, mucolysin A; HetA, heterolysin A; Δ37VerA, deletion mutant of versicolysin A; MW, molecular weight; kDa, kilo Dalton; Ip, isoelectric point.

Table S3. Basic biochemical characteristics of native MACPF proteins analyzed in the study.

	Number of amino acids	MW (kDa)	Ip	% Amino acid identity with PlyB
Δ48PlyB	476	52.2	6.4	100
Δ30NudB	475	53.1	5.7	71.7
Δ43MucB	491	54.0	5.6	70
Δ21HetB	474	52.0	6.1	61.2
Δ29VerB	477	52.4	4.9	57.3

Δ48PlyB, deletion mutant of pleurotolysin B; Δ30NudB, deletion mutant of nudolysin B; Δ43MucB, deletion mutant of mucolysin B; Δ21HetB, deletion mutant of heterolysin B; Δ29VerB, deletion mutant of versicolysin B; MW, molecular weight (kDa); Ip, isoelectric point; MACPF, membrane attack complex/perforin.

Table S4. The amounts of immobilized vesicles in RU units for each SPR experiment.

pH	Aegerolysin	Sf9	CPE: POPC:Chol (1:1:1)	RBC	SM:Chol (1:1)	PS: POPC: Chol (1:1:1)	PE: POPC: Chol (1:1:1)	CL: POPC: Chol (1:1:1)	PA: POPC: Chol (1:1:1)	POPC: Chol (1:1)
6	OlyA6	5200	6500	1000	9500	4100	9000	7400	5500	8400
	HetA	5200	6500	1000	9500	4100	9000	7400	5500	8400
	NudA	5200	6500	1000	9500	4100	9000	7400	5500	8400
7	OlyA6	5500	10000	3800	12000	6000	8200	6800	7100	8000
	HetA	5500	10000	3800	12000	6000	8200	6800	7100	8000
	NudA	5200	7000	3800	8800	4700	8200	4100	5600	8000
8	OlyA6	4800	6000	1000	9000	3800	7800	3500	5000	8500
	HetA	4800	6000	1000	9000	3800	7800	3500	5000	8500
	NudA	4800	6000	1000	9000	3800	7800	3500	5000	8500

OlyA6, ostreolysin A6; HetA, heterolysin A; NudA, nudolysin A; Sf9, lipid isolate of *Spodoptera frugiperda* Sf21 cells; CPE, ceramide phosphoethanolamine; POPC; 1-palmitoyl-2-oleoyl-*sn*-glycero-3-phosphocholine; Chol, cholesterol; RBC, lipid isolate from bovine erythrocyte; SM, sphingomyelin; PS, phosphatidylserine; PE, phosphatidylethanolamine; CL, cardiolipin; PA, phosphatidic acid; SPR, surface plasmon resonance; RU, response unit.

Table S5. The list of primers used in the study.

Name	Sequence (5'-3')	Gene amplification
F-HetA	TTT TTT CAT ATG GCT TAC GCT CAG TGG G	HetA
R-HetA	TTT TTT CTC GAG GCT ACC GCG AGG	
F-HetB	TTT TTT GGA TCC ATT GCT GGA GAC ACG	HetB
R-HetB	TTT TTT ACG CGT TTA CTC TTC ACC AGA	
Δ37VerA-F	TTT TTT CAT ATG CGT GCG TAC GCG CAG TG	Δ37VerA
Δ37VerA-R	TTT TTT CTC GAG GCC TTT ATA CAC GGT TTC	
F-NudA	TTT TTT CAT ATG GCG TAT GCG CAG TG	NudA
R-NudA	TTT TTT CTC GAG GCC GCT GCC	
F-NudB	TTT TTT CAT ATG GGC AGC GAA GGC AAA	NudB
R-NudB	TTT TTT GGA TCC TTA CTC CTC GTT AGA GT	
F-MucA	TTT TTT CAT ATG GCG TAC GCA CAA TG	MucA
R-MucA	TTT TTT CTC GAG GCC GCT GCC	
F-MucB	TTT TTT CAT ATG GGC AGC GAA GGC AAA	MucB
R-MucB	TTT TTT CTC GAG TTA CTC CTC GCG GG	

F, forward; R, reverse; HetA, heterolysin A; HetB, heterolysin B; Δ37VerA, deletion mutant of versicolysin A; NudA, nudolysin A; NudB, nudolysin B; MucA, mucolysin A; MucB, mucolysin B.

Table S6. The list of proteins, corresponding vectors, cloning sights, and sizes of expressed proteins.

Protein	Vector and antibiotic resistance	Restriction sights	Size of the expressed proteins (kDa)
HetA	pET21c(+) (Ap)	*Nde*I and *Xho*I	17.5
HetB	pET8c(+) (Ap)	*Bam*HI and *Mlu*I	53.5
VerA	pET21b(+) (Ap)	*Nde*I and *Xho*I	20.1
VerB	pET28a(+) (Kn)	*Nde*I and *Xho*I	54.5
Δ37VerA	pET21b(+) (Ap)	*Nde*I and *Xho*I	16.1
NudA	pET21c(+) (Ap)	*Nde*I and *Xho*I	17.7
NudB	pET19b(+) (Ap)	*Nde*I and *Bam*HI	57.3
MucA	pET21c(+) (Ap)	*Nde*I and *Xho*I	15.8
MucB	pET28a(+) (Kn)	*Nde*I and *Xho*I	57.0

HetA, heterolysin A; HetB, heterolysin B; VerA, versicolysin A; VerB, versicolysin B; Δ37VerA, deletion mutant of versicolysin A; NudA, nudolysin A; NudB, nudolysin B; MucA, mucolysin A; MucB, mucolysin B; Ap, ampicilin; Kn, kanamycin; kDa, kilo Dalton.

Table S7. Signal peptides prediction.

Aegerolysin	Prediction	Other	Sec/SPI
OlyA6	No SP	0.8345	0.1655
NudA	No SP	1	0
MucA	No SP	1	0
HetA	No SP	1	0
VerA	No SP	1	0
Partner protein			
PlyB	No SP	1	0
NudB	No SP	1	0
MucB	No SP	1	0
HetB	No SP	1	0
VerB	No SP	1	0

The SignalP 6.0 algorithm predicts signal peptides and the location of their cleavage sites using a machine learning model in proteins from Eukarya [37]. Sec/SPI - "standard" secretion signal peptides are transported by the Sec translocon and cleaved by signal peptidase I. OlyA6, ostreolysin A6; NudA, nudolysin A; MucA, mucolysin A; HetA, heterolysin A; VerA, versicolysin A; PlyB, pleurotolysin B; NudB, nudolysin B; MucB, mucolysin B; HetB, heterolysin B; VerB, versicolysin B; NO SP, no signal peptide; Sec/SPI, Sec translocon signal peptidase I.

Table S8. Protein localization prediction.

**Localiza-tion**	Aegerolysins					MACPF partner proteins					Thresh-old
	OlyA6	NudA	MucA	HetA	VerA	PlyB	NudB	MucB	HetB	VerB	
**C**	0.6021	**0.6107**	0.5291	0.5430	0.6027	0.4151	0.5263	0.4468	0.4872	0.5240	0.4761
N	0.4953	0.4735	0.5495	0.3387	0.4169	0.2215	0.2894	0.2470	0.2729	0.2334	0.5014
E	**0.7932**	**0.7508**	0.6460	**0.7606**	**0.7898**	**0.7000**	0.5695	0.6129	0.6572	0.5314	0.6173
CM	0.3246	0.3137	0.2558	0.2373	0.1400	0.4094	0.3962	0.3931	0.4038	0.5039	0.5646
M	0.2421	0.2370	0.3029	0.3169	0.1926	0.1224	0.1174	0.1183	0.1141	0.0762	0.6220
PL	0.0030	0.0041	0.0102	0.0160	0.0520	0.0598	0.0419	0.0677	0.0970	0.1018	0.6395
ER	0.0718	0.0949	0.0554	0.0492	0.0740	0.0748	0.0717	0.1136	0.0861	0.0747	0.6090
L/V	0.0963	0.1597	0.1202	0.1914	0.1658	0.2067	0.1544	0.1564	0.1633	0.1502	0.5848
GA	0.1402	0.2194	0.3174	0.1876	0.1387	0.2461	0.2300	0.2560	0.3056	0.2537	0.6494
PE	0.0087	0.0091	0.0217	0.0405	0.0782	0.2543	0.2513	0.2583	0.2080	0.1342	0.7364

The presence or absence of a signal peptide does not say everything about the localization of eukaryotic proteins. The prediction program for the subcellular localization **DeepLoc 2.0,** can find more about the sorting of these proteins [38]. OlyA6, ostreolysin A6; NudA, nudolysin A; MucA, mucolysin A; HetA, heterolysin A; VerA, versicolysin A; PlyB, pleurotolysin B; NudB, nudolysin B; MucB, mucolysin B; HetB, heterolysin B; VerB, versicolysin B; C, cytoplasm; N, nucleus; E, extracellular; CM, cell membrane; M, mitochondrion; PL, plastid; ER, endoplasmic reticulum; L/V, lysosome/vacuole; GA, Golgi apparatus; PE, peroxisome; MACPF, membrane attack complex/perforin.

3. Supplementary figures

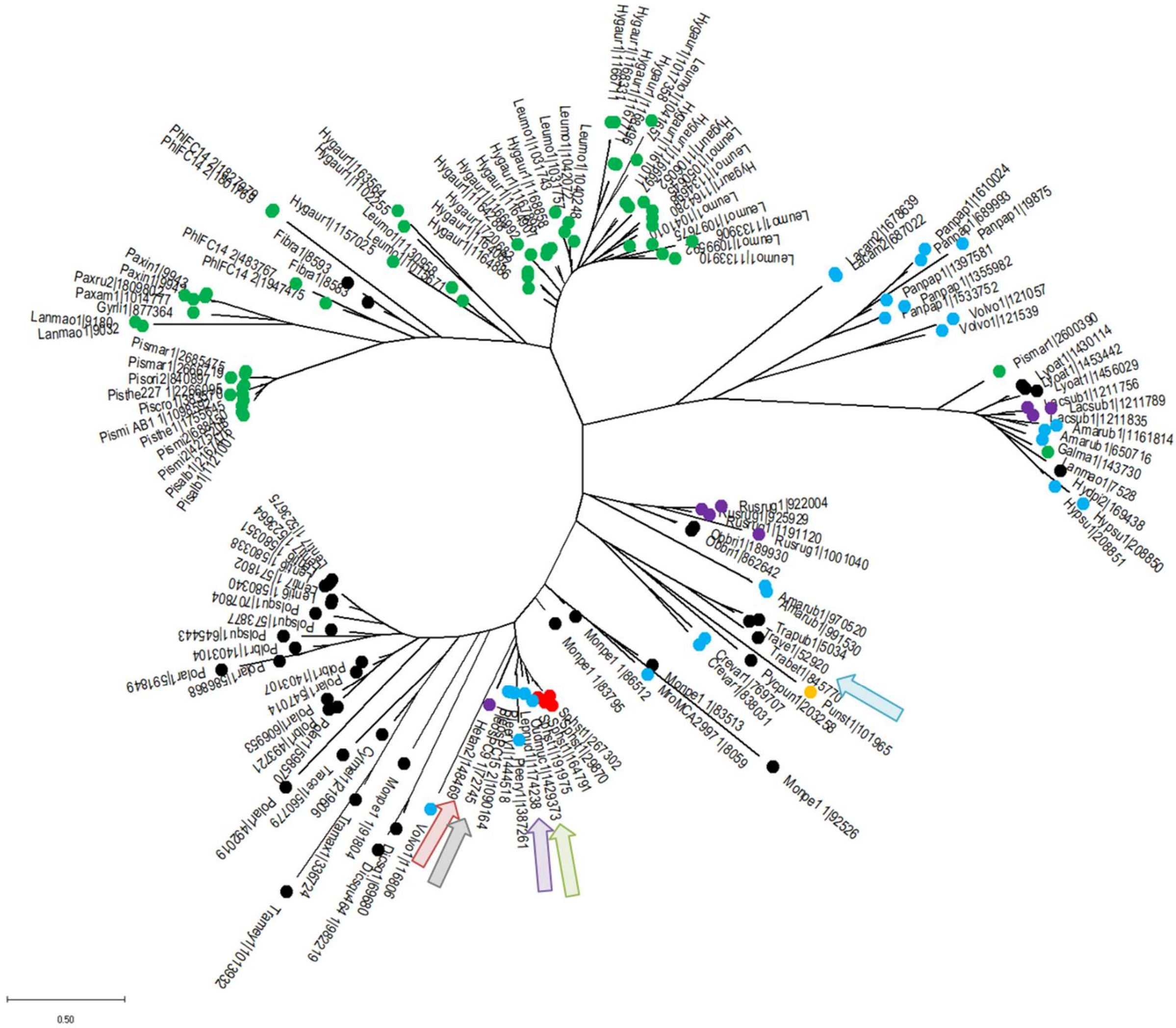

Figure S1. Phylogenetic analysis of aegerolysins from Agaricomycetes. Aegerolysins (160) from published fungal genomes (60) identified in the JGI MycoCosm database [79] by Pfam protein domain PF06355 (**Table S1**). After muscle alignment

of the sequences (139), maximum likelihood phylogenetic tree was inferred using the MEGA tools [26,27]. Aegerolysins belonging to different orders of *Agaricomycetes* [25] are marked with closed colored circles: *Agaricales*, blue; *Boletales*, green; *Corticiales*, orange; *Geastrales*, red; *Polyporales*, black; and *Russulales*, purple. Aegerolysins from selected fungal species are indicated with arrows: pleurotolysin A from *P. ostreatus* PC9 (PleosPC9_1|72745, grey), heterolysin A from *H. irregulare* (Hetan2|148469, ruby), nudolysin A from *L. nuda* CBS 247.69 (Lepnud1|1174238, violet purple), versicolysin A from *T. versicolor* (Trave1|52920, deep teal), and mucolysin A from *M. mucida* (CBS 558.79, Oudmuc1|1429373, green).

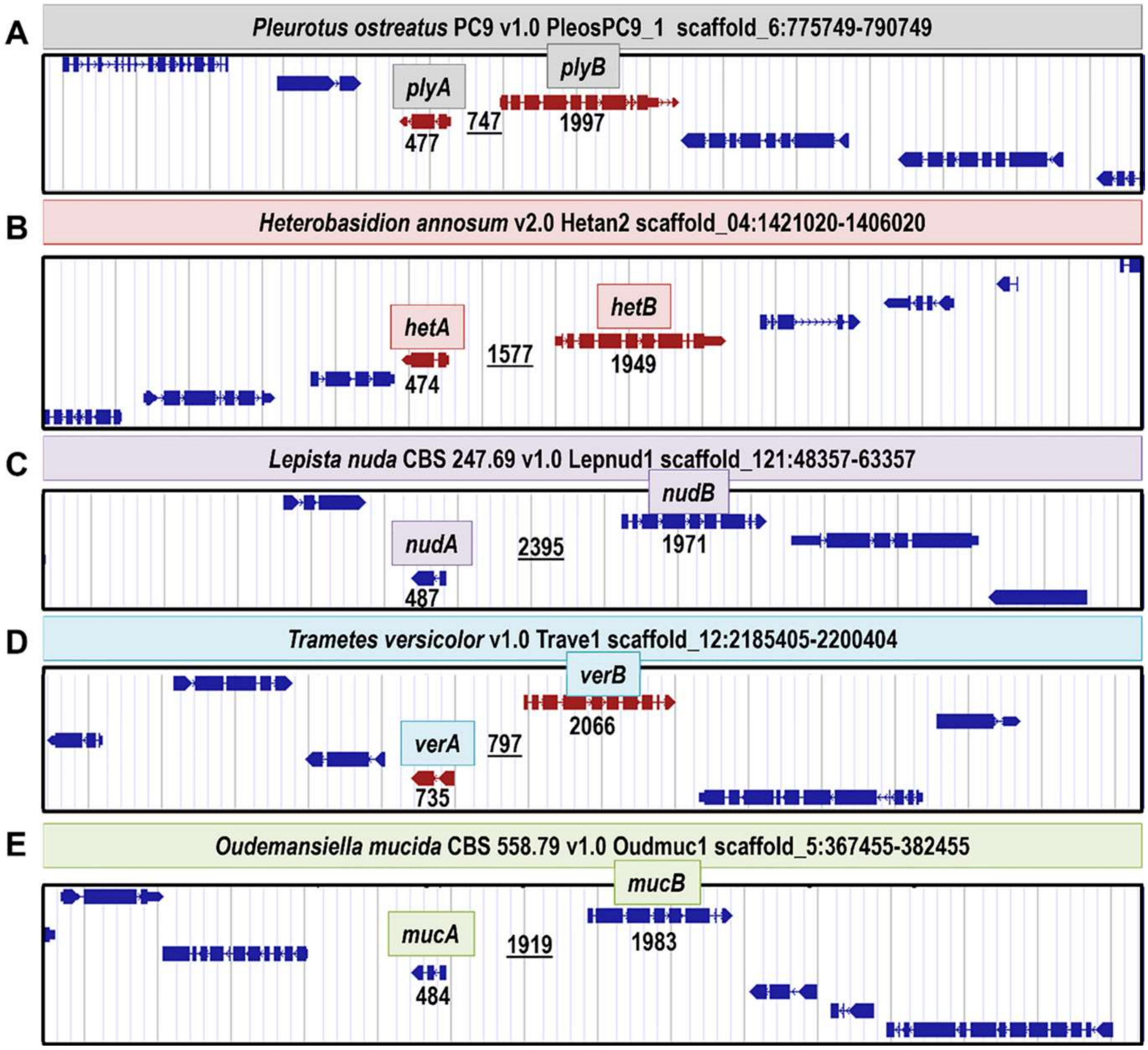

Figure S2. Aegerolysin gene loci in fungal genomes. Aegerolysin and MACPF genes: *plyA* and *plyB*, pleurotolysin A and B from *Pleurotus ostreatus PC9 v1.0* genome; *hetA* and *hetB*, heterolysin A and B from *Heterobasidion annosum* v2.0 (*H. irregulare*) genome; *nudA* and *nudB*, nudolysin A and B from *Lepista nuda* CBS 247.69 v1.0; *verA* and *verB*, versicolysin A from *Trametes versicolor* v1.0; and *mucA* and *mucB*, mucolysin A and B from *Oudemansiella mucida* CBS 558.79 v1.0 (*Mucidula mucida*) from JGI MycoCosm database [79]; blue, automatically annotated genes; red, manually annotated genes; number, gene size in base pairs from the first to the last coding codon; underlined number, inter-gene distance; MACPF, membrane attack complex/perforin.

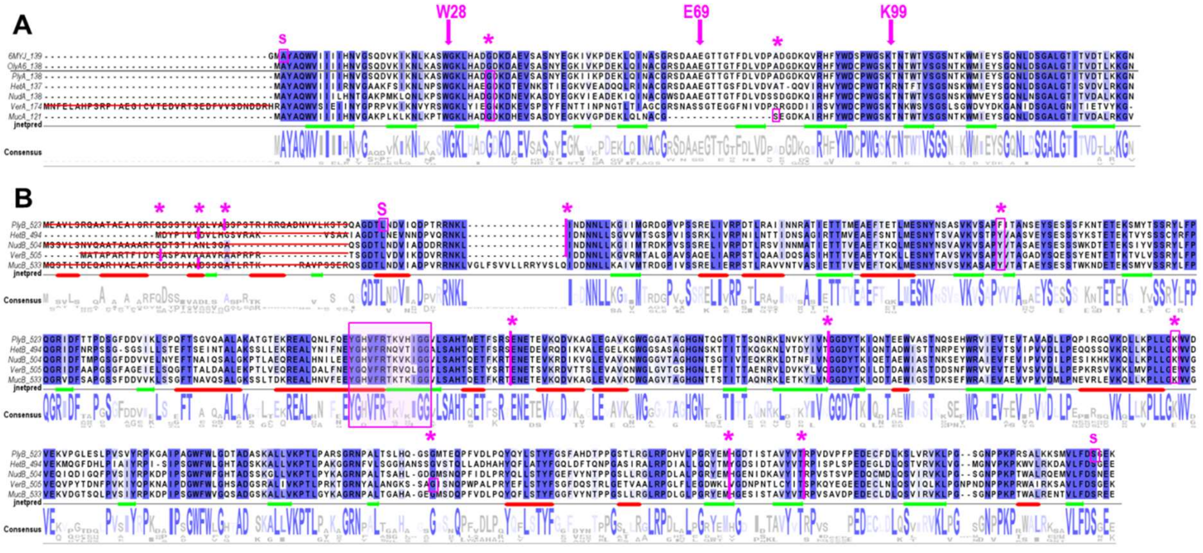

Figure S3. Alignments of amino acid sequences and prediction of secondary structures of selected aegerolysins and MACPF proteins. A multiple sequence alignment was performed using the muscle algorithm and presented by Jalview [52]. The alignment is colored according to percent of identity (blue, >50 %,); the consensus sequence is shown below; magenta asterisk, arrow or box, position of the intron at the protein level); magenta s and box, start and end of the PlyB sequence from PDB ID: 4OEJ structure. The secondary structures of the proteins were predicted using the JPred4 server, which is accessible via Jalview [52]. JNetPRED consensus prediction for 6MYJD and PlyB sequences, respectively: green sheets, and red helices. Red strikethrough, N-terminus sequence omitted from the expression constructs. (**A**) PDB ID: 6MYJD–OlyA6–PlyA, ostreolysin A6–pleurotolysin A from *P. ostreatus*; HetA, heterolysin A from *H. irregulare*; NudA, nudolysin A from *L. nuda*; VerA, versicolysin A from *T. versicolor*; and MucA, mucolysin A from *M. mucida*. The positions of the amino acids involved in SM binding in the PDB ID–6MYJD structure are numbered [32]; SM, sphingomyelin. (**B**) Partner proteins: PlyB, pleurotolysin B; HetB, heterolysin B; NudB, nudolysin B; VerB, versicolysin B; and MucB, mucolysin B. Larger magenta box, typical signature Y/F-G-X_2-F/Y-X_6-G-G for the MACPF domain in fungi, [29]; MACPF, membrane attack complex/perforin.

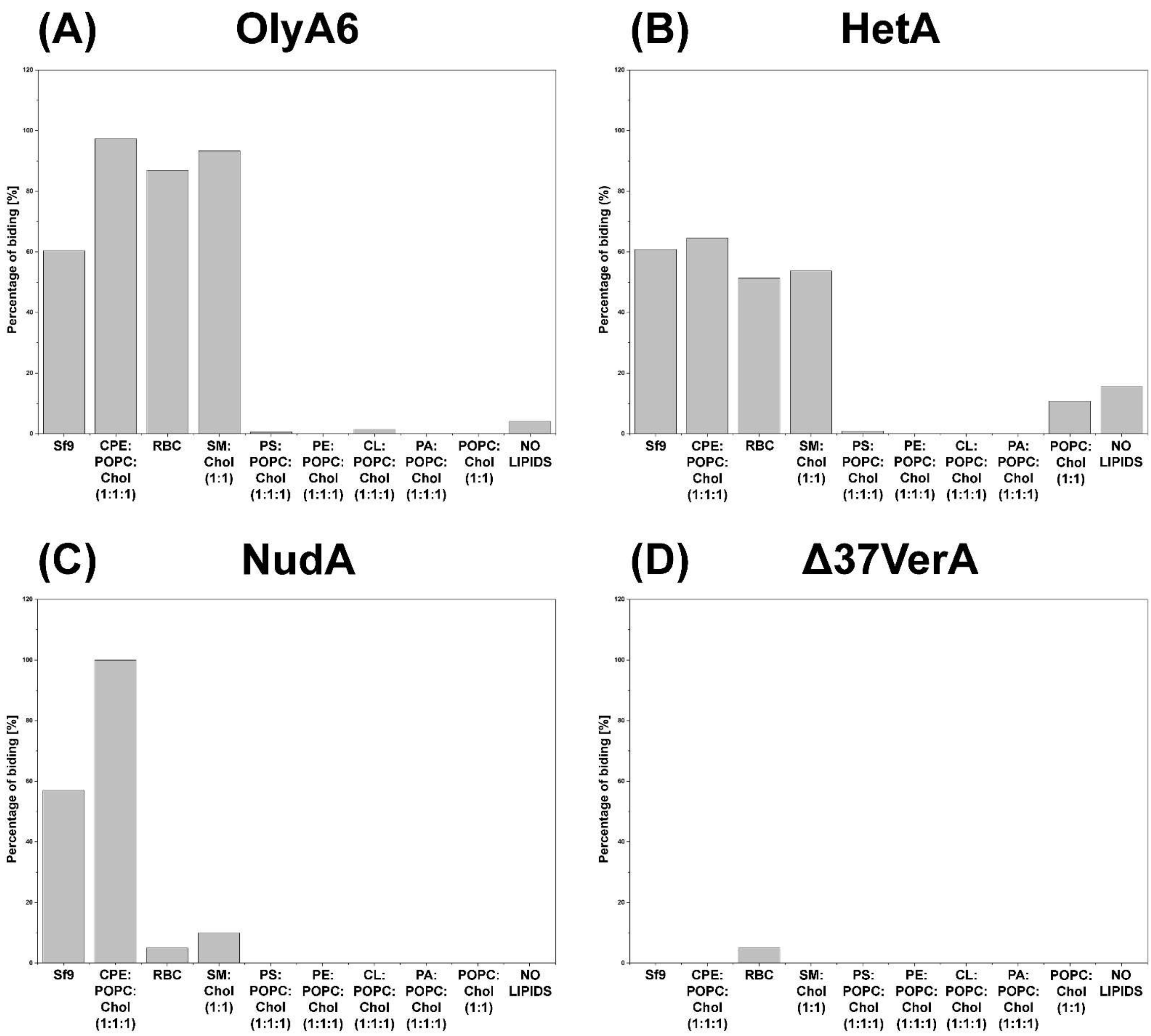

Figure S4. Assessment of OlyA6, HetA, NudA and Δ37VerA association with lipid vesicles at pH 8.0. SDS-PAGE of pellets (membrane-bound proteins) collected after MLV sedimentation assays. Quantification of MLV sedimentation assay results using GelQuantNET. Representative data of triplicates are shown. OlyA6, ostreolysin A6; HetA, heterolysin A; NudA, nudolysin A, Δ37VerA, truncated version of versicolysin A; Sf9, total lipid extract from *Spodoptera frugiperda* Sf21 cells; CPE, ceramide phosphoethanolamine; POPC, 1-palmitoyl-2-oleoyl-*sn*-glycero-3-phosphocholine; Chol, cholesterol; RBC, total lipid extract form bovine erythrocytes; SM, sphingomyelin; PS, phosphatidylserine; PE, phosphatidylethanolamine; CL, cardiolipin; PA; phosphatidic acid. Representative data of triplicate experiments, in which the standard error did not exceed 5%, are shown.

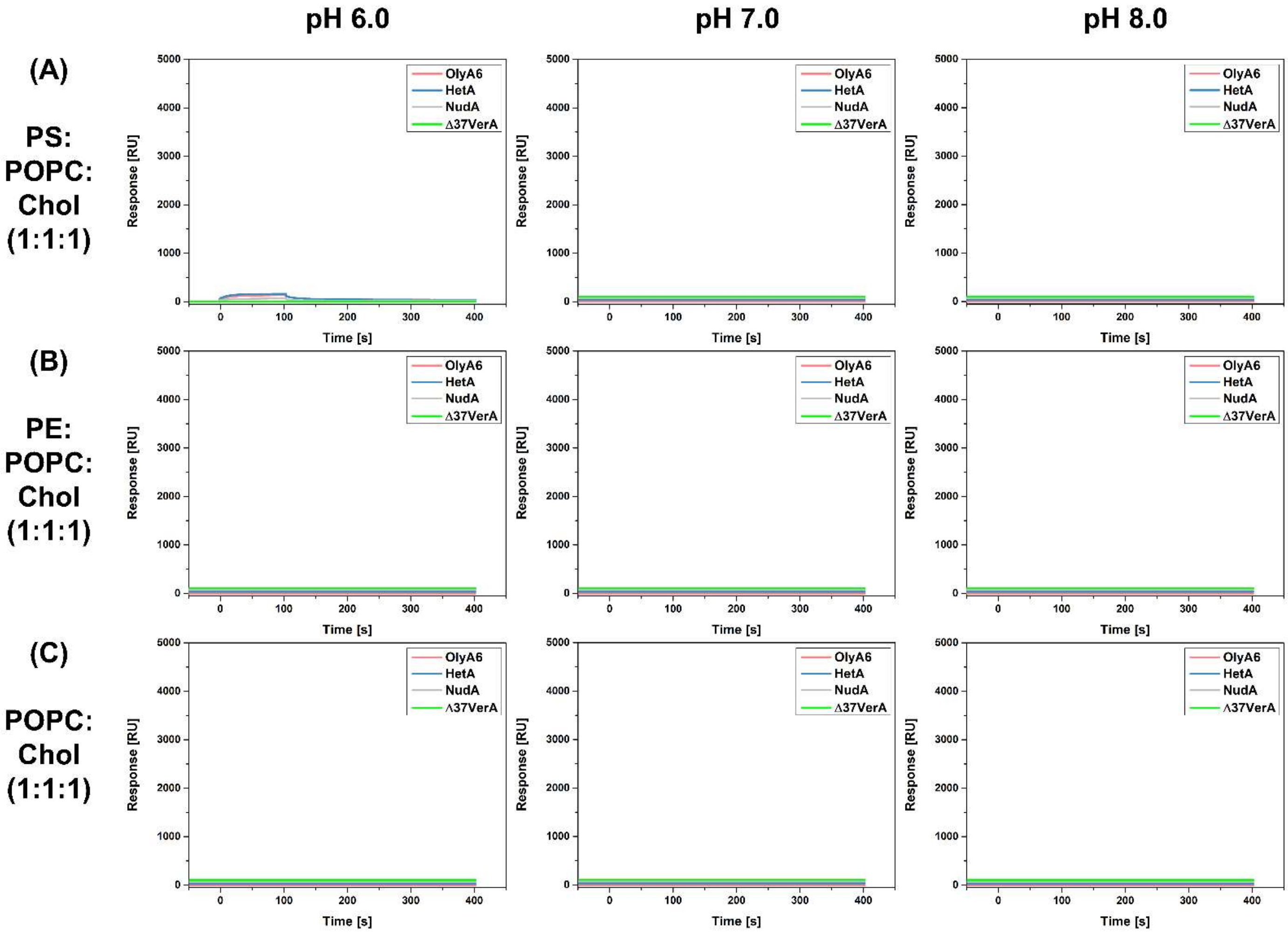

Figure S5. Surface plasmon resonance analysis of interaction of aegerolysins with LUVs containing glicerophospholipid receptors. Large unilamellar vesicles (LUVs) containing PS (**A**), PE (**B**), or only POPC and Chol (**C**) were immobilized on a Biacore L1 chip to 3.500 – 9.000 RU and analytes (OlyA6, NudA, HetA and Δ37VerA in the 5 μM concentration) injected at a flow rate of 10 μL/min with association time 1 min in a running buffer (pH 6.0, 7.0, or 8.0) at 25 °C. Representative sensorgrams of triplicates are shown. OlyA6, ostreolysin A6; HetA, heterolysin A; NudA, nudolysin A; Δ37VerA, deletion mutant of versicolysin A; POPC; 1-palmitoyl-2-oleoyl-*sn*-glycero-3-phosphocholine; Chol, cholesterol; PS, phosphatidylserine; PE, phosphatidylethanolamine; SPR, surface plasmon resonance.

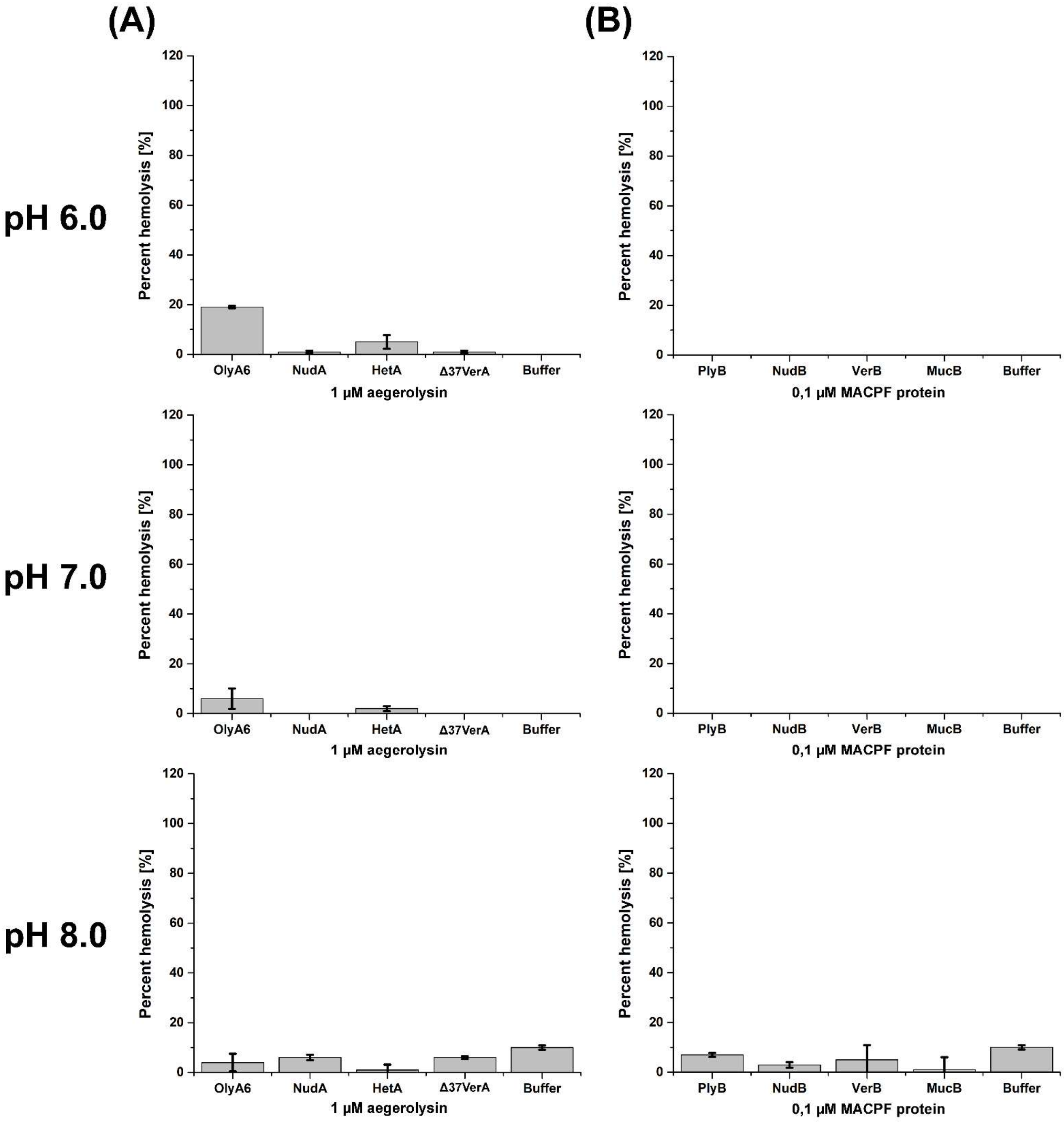

Figure S6. Hemolytic activity of 1 µM aegerolysins or 0.1 µM MACPF proteins at pH 6.0, 7.0, and 8.0. Percentage of hemolysis of RBC cells treated with 1 µM OlyA6, NudA, HetA, or Δ37VerA, or with 0.1 µM PlyB, NudB, VerB, or MucB, is expressed as the ratio of absorbances (630 nm) of RBC at the beginning (time = 0) and at the end of the experiment (time = 60 min) x 100. OlyA6, ostreolysin A6; HetA, heterolysin A; NudA, nudolysin A, Δ37VerA, truncated version of versicolysin A; PlyB, pleurotolysin B; NudB, nudolysin B, VerB versicolysin B; MucB, mucolysin B; RBC; bovine red blood cells; MACPF, membrane attack complex/perforin.

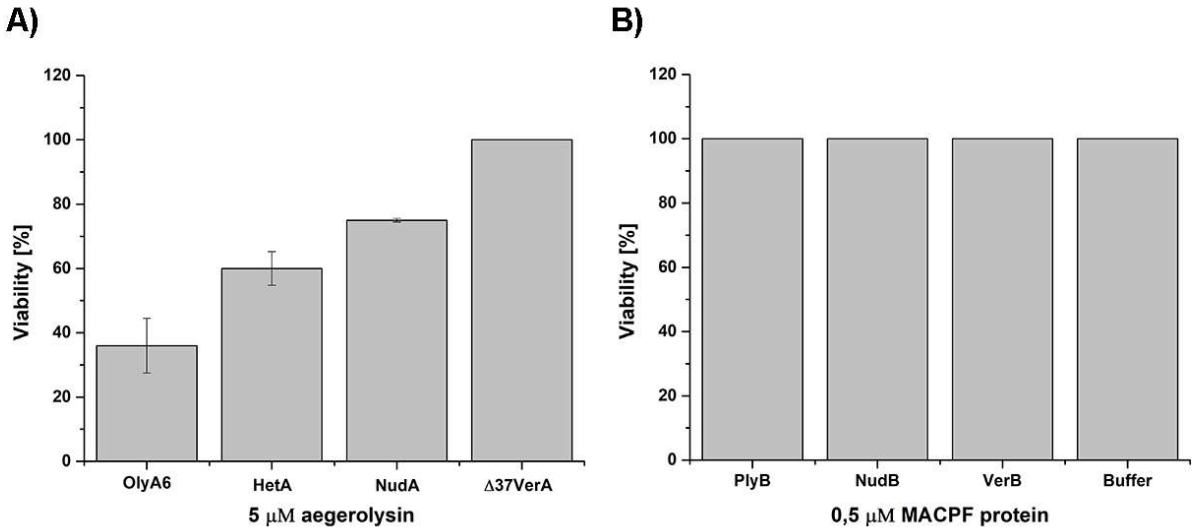

Figure S7. Effect of aegerolysins and their native MACPF protein partners alone on survival of Sf9 cells. Percentage of viability in % of Sf9 cells treated with 5 μM OlyA6, HetA, NudA, or Δ37VerA or 0.5 μM PlyB, NudB, or VerB is expressed as absorbance of treated cells/ absorbance of control cells × 100 % after 1 h exposure. Data are means ± SD from three independent experiments. OlyA6, ostreolysin A6; HetA, heterolysin A; NudA, nudolysin A, Δ37VerA, truncated version of versicolysin A; PlyB, pleurotolysin B; NudB, nudolysin B; VerB versicolysin B; Sf9, *Spodoptera frugiperda*; MACPF, membrane attack complex/perforin.
